# Electrocatalytic Properties of Ni(II) Schiff Base Complex Polymer Films

**DOI:** 10.3390/ma15010191

**Published:** 2021-12-28

**Authors:** Danuta Tomczyk, Wiktor Bukowski, Karol Bester, Michalina Kaczmarek

**Affiliations:** 1 Department of Inorganic and Analytical Chemistry, University of Łódź, ul. Tamka 12, 91-403 Lodz, Poland; michalina.kaczmarek@unilodz.eu; 2 Faculty of Chemistry, Rzeszów University of Technology, Al. Powstańców W-wy 6, 35-959 Rzeszow, Poland; wbuk@sd.prz.edu.pl (W.B.); bester_k@prz.edu.pl (K.B.)

**Keywords:** thin and thick films, salen, nickel complex, modified electrodes, electrocatalysis

## Abstract

Platinum electrodes were modified with polymers of the (±)-*trans*-N,N′-bis(salicylidene)-1,2-cyclohexanediaminenickel(II) ([Ni(salcn)]) and (±)-*trans*-N,N′-bis(3,3′-*tert*-Bu-salicylidene)-1,2-cyclohexanediaminenickel(II) ([Ni(salcn(Bu))]) complexes to study their electrocatalytic and electroanalytical properties. *Poly*[Ni(salcn)] and *poly*[Ni(salcn(Bu))]) modified electrodes catalyze the oxidation of catechol, aspartic acid and NO_2_^−^. In the case of *poly*[Ni(salcn)] modified electrodes, the electrocatalysis process depends on the electroactive surface coverage. The films with low electroactive surface coverage are only a barrier in the path of the reducer to the electrode surface. The films with more electroactive surface coverage ensure both electrocatalysis inside the film and oxidation of the reducer directly on the electrode surface. In the films with the most electroactive surface coverage, electrocatalysis occurs only at the polymer–solution interface. The analysis was based on cyclic voltammetry, EQCM (electrochemical quartz crystal microbalance) and rotating disc electrode method.

## 1. Introduction

Complexes of Ni (II) and other transition metals with Schiff bases of the N_2_O_2_ type have been studied for a long time and are still under investigation. One of the reasons for interest is the use of these complexes in homogeneous electrocatalysis. They were used, among others, to reduce alkyl and aryl halides [1], proton reduction [2,3] and oxidation of cycloalkenes [4]. They are used in the asymmetric catalysis of many organic compounds [5] and in the epoxidation of olefins [6].

Another reason for interest is the possibility of receiving electroactive polymer films on the electrode surfaces, which are used in electrocatalysis and electroanalysis [7,8,9,10,11,12,13,14,15,16,17,18]. Such modified electrodes can be used as oxidizing agents of methanol, ethanol, hydrazine, glycerol [7,8] and ferrocene [9]. They catalyze the reduction of alkyl and aryl halides [10], hydrogen peroxide and oxygen [11] and propylene dimerization [12]. The relative ease of obtaining such electrodes allows their use as sensors, e.g., dipyrone [13], barium ions [14], nitric oxide [15] and BrO_3_^−^, IO_3_^−^, IO_4_^−^ anions [16], furosemide [17] and oxygen [18]. They also play the role of potential materials in energy storage devices [19,20,21,22,23,24,25].

Electrodes modified with salen type ligand complexes are obtained by anodic electropolymerization, carried out in a solution of the complex in a solvent with poor coordinating properties [26,27,28,29,30,31,32,33,34]. Conductivity of polymer films is ensured by charge delocalization through polymer chains formed by *phenyl*–*phenyl* [26,27,28,29,30,31] or π-stacking interactions [32,33]. The mechanism of electropolymerization is still under discussion. It can be based on a ligand [26,27,28,29,30,31], a central metal ion [32,33] or both a ligand and central ion [34].

Our research presents electrodes modified with polymers of Ni (II) complexes with (±)-*trans*-N,N′-bis(salicylidene)-1,2-cyclohexanediamine (*poly*[Ni(salcn)]) and its *tert*-Bu derivative, substituted in ortho positions of phenolate moieties (*poly*[Ni(salcn(Bu))]). In previous studies, we have shown that they are conductive polymers [35]. They oxidize in two steps to the *macro*phenoxyl radical and *macro*bisphenoxyl radical [36,37]. The research also showed the influence of the substituent and the electroactive surface coverage on the kinetics of electrode processes [38].

The purpose of the present work was to test the modified electrodes for their application in electrocatalysis. The investigations were carried out in CH_2_Cl_2_, AN (acetonitrile) and H_2_O solutions. The choice of CH_2_Cl_2_ and AN was imposed by the fact that these solvents are media ensuring very good stabilization of *poly*[Ni(salcn)] modified electrodes. *Poly*[Ni(salcn(Bu))] modified electrodes are most stable in CH_2_Cl_2_. Frequently studied reducing agents were selected for investigation due to their biological functions or environmental impact (catechol, ascorbic acid and NO_2_^−^) [39,40,41,42]. The analysis of the electrocatalysis process was performed with ferrocene as a reducing agent with well-known and stable electrochemical characteristics.

## 2. Materials and Methods

### 2.1. Chemicals

(±)-*trans*-N,N′-bis(salicylidene)-1,2-cyclohexanediaminenickel(II) [Ni(salcn)] and (±)-*trans*-N,N′-bis(3-*tert*-butylsalicylidene)-1,2-cyclohexanediaminenickel(II) [Ni(salcn(Bu))] complexes have been synthesized according to the previously presented procedure [36]. Salicylaldehyde, 99% and (±)-*trans*-1,2-diaminocyclohexane were obtained from Merck-Sigma-Aldrich (Darmstadt, Germany), and nickel(II) acetate tetrahydrate Ni(AcO)_2_·4H_2_O (reagent grade) was purchased from POCh. (Gliwice, Poland).

Methylene chloride (CH_2_Cl_2_) and acetonitrile (AN), HPLC grade, were obtained from Baker, and ethanol (EtOH) 95% was from POCh (Gliwice, Poland). Tetrabutylammonium hexafluorophosphate (TBAH), analytical grade, was obtained from Fluka. Catechol 99%, ascorbic acid 99% and ferrocene 98% were obtained from Sigma. Sodium perchlorate 98%, sodium hydroxide 98% and sodium nitrate(III) 98% were purchased from POCh (Gliwice, Poland). All of them were used as received.

### 2.2. Modification of Electrodes

**Scheme 1:**
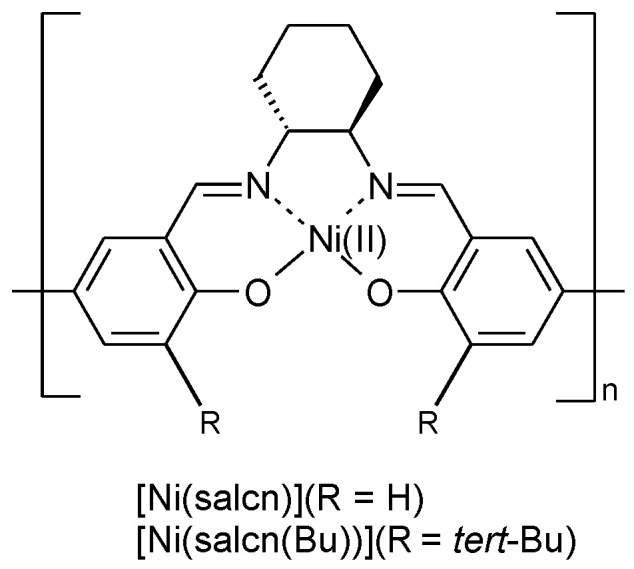
Structural formula of polymers modifying the platinum electrode.

The platinum disk electrode (MINERAL, Warsaw, Poland) was modified with polymers of [Ni(salcn)] and [Ni(salcn(Bu))] complexes (Scheme 1).

The platinum disk electrode before measurements was cleaned in aqueous suspension of 0.05 μm alumina micropolish. Polymers were obtained by anodic electropolymerization using cyclic voltammetry. Curves were recorded in solutions of complexes (10^−3^ mole·dm^−3^) in CH_2_Cl_2_/TBAH (0.1 mole·dm^−3^), at scan rates of 0.005–0.5 V·s^−1^ in positive potential ranges (0–1.6 V), involving two-step oxidation of complexes [35,37]; 1 to 20 electropolymerization cycles were recorded. As a result of the electropolymerization process, yellow electroactive, well adhering to the electrode surface polymer films, were obtained.

The thickness of the polymer films was regulated by the number of electropolymerization cycles as well as the speed of electrode polarization due to the lack of reversibility of the electrode processes. Using lower scan rates, electrodes were obtained modified with a larger amount of adsorbed film, due to the longer duration of the electrode process.

### 2.3. Measurements of Modified Electrodes

Modified electrodes were tested by cyclic voltammetry, by the rotating disc electrode method (RDE) and by the EQCM method.

Electroactive surface coverage (*Γ*) was determined on the basis of voltammograms of electrodes modified in the electrolyte solution, recorded at *v* = 0.01 V·s^−1^, based on Faraday’s law, *Γ* = *Q*/*zFA,* where *Q* is the charge passed in the electrode process, *z* is the number of electrons involved in an electrode reaction, *F* is the Faraday constant and *A* is the electrode surface. The low electrode polarization rate was used to obtain the conditions enabling the oxidation of the entire film.

The investigation of modified electrodes were carried out in CH_2_Cl_2_/TBAH (0.1 mol·dm^−3^), AN/TBAH (0.1 mol·dm^−3^) and H_2_O/NaClO_4_/NaOH (0.1 mol·dm^−3^) solutions, in the potential range corresponding to a two-step polymer film oxidation process (0–1.6 V) and in solutions of abovementioned electrolytes containing catechol (10^−3^ and 5 × 10^−3^ mol·dm^−3^), ascorbic acid (10^−3^ mol·dm^−3^), NO_2_^−^ (4 × 10^−4^–5 × 10^−3^ mol·dm^−3^) and ferrocene (2 × 10^−4^ and 10^−3^ mol·dm^−3^) reducing agents. The tests were carried out at the polarization rate of the working electrode, *v* = 0.05 V·s^−1^, vs. Ag/AgCl (in 1 mol·dm^−3^ KCl), connected to the bulk of the solution by a Luggin capillary. The auxiliary electrode was a platinum wire. The cyclic voltammetry and rotating disc electrode method were performed using AUTOLAB PGSTAT 10 Eco Chemie. EQCM measurements were carried out using a module Autolab Electrochemical Quartz Crystal Microbalance (Metrohm-Autolab, Utrecht, The Netherlands), fitted with 6 MHz, AT-cut crystals coated with Pt, with the AUTOLAB PGSTAT 302N, controlled by NOVA software (version 1.11.1). The measurement procedure included electropolymerization of the film from the complex solution by cyclic voltammetry on a quartz microbalance. After this process, the film was washed with solvent, conditioned in solvent for 5 min to remove as much free complex as possible from the film structures, and then conditioned in a stock electrolyte solution to establish ion equilibrium in the neutral film. For the film prepared in this way, cyclic voltammetry was carried out on a quartz microbalance in a supporting electrolyte solution or in ferrocene. Data analysis was performed based on the Koutecky–Levich equation, 1/*i_l_* = 1/0.62*zFAc_s_D_s_^2/3^υ*^−*1/6*^*ϖ^1/2^* + 1/*nFAk’c_s_*, where *i_l_* is limiting current, *c_s_* is the solute bulk concentration, *D_s_* is the diffusion coefficient of the substrate in solution, *υ* is the kinematic viscosity of the solution, *ϖ* is rotation rate of the electrode and *k’* is the effective heterogeneous rate constant for the modified electrode. All studies were performed under an argon atmosphere at room temperature. The values of the potentials are reported relative to the Ag/AgCl (1 mol·dm^−3^ KCl) reference electrode.

## 3. Results and Discussion

### 3.1. Processes Occurring on Modified Electrodes in the Supporting Electrolytes

**Figure 1:**
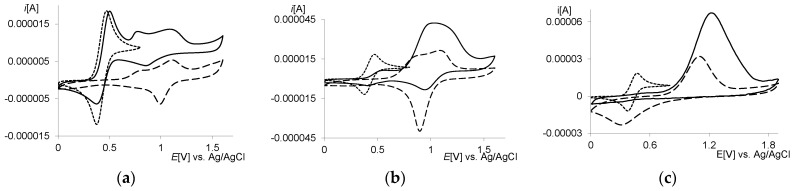
Cyclic voltammograms, 2nd scans, *v* = 0.05 V·s^−1^, vs. Ag/AgCl. Pt*poly*[Ni(salcn)] in TBAH (0.1 mol·dm^−3^)/CH_2_Cl_2_—dashed lines, Pt*poly*[Ni(salcn)] in ferrocene (10^−3^ mol·dm^−3^)/TBAH/CH_2_Cl_2_—solid lines; Pt in ferrocene/TBAH/CH_2_Cl_2_—dotted lines: (**a**) after electropolymerization: 3 scans, *v* = 0.5 V·s^−1^; (**b**) after electropolymerization: 3 scans, *v* = 0.05 V·s^−1^; (**c**) after electropolymerization: 3 scans, *v* = 0.005 V·s^−1^.

In the case of *poly*[Ni(salcn)] in CH_2_Cl_2_ and AN solutions, the oxidation process of the polymer deposited on the electrode surface depends on the electroactive surface coverage [37]. In CH_2_Cl_2_ solutions, for the films with less electroactive surface coverage, a two-step oxidation process was observed, at 0.78 V and at 1.12 V (Figure 1a, dashed line). This process corresponds to the oxidation of the ligand phenolate groups successively to phenoxyl radicals and bisphenoxyl radicals, which was shown earlier on the basis of spectroelectrochemical studies [37]. The observed difference of the anode peaks potentials was (Δ*E_II–I_* = 0.34 V). However, as the electroactive surface coverage increased, the potential of the first step of the process increased (*E_I_* = 0.83 V) (Figure 1b, dashed line), and the differences of the anode peaks potentials decreased (Δ*E_II–I_* = 0.28 V) as a result of a decrease in relocation [43], caused by an increase in difficulties in the charge transfer [38]. However, in the films with the most concentration of active centers, the anode process became one-step and occurs at the potential corresponding to the peak potential of the second step of oxidation of *poly*[Ni(salcn)] (*E* = 1.12 V) (Figure 1c, dashed line). Limitations in delocalization, the number of steps in the anode process and in kinetics, proceeding with the increase in the electroactive surface coverage, are most likely related to the cross-linked structure of this polymer, as shown by FTIR ATR spectroscopy studies [35]. The measure of difficulties in charge transfer are lower values of *cD*^1/2^ (*D*—diffusion coefficient) for films of this polymer than for *poly*[Ni(salcn(Bu))] films, while the concentration of active surface centers (*Γ*) in *poly*[Ni(salcn(Bu))] are smaller than in the *poly*[Ni(salcn)] films (the comparison for polymers obtained by recording the same number of electropolymerization cycles and at the same electrode polarization rate) [38]. The *poly*[Ni(salcn)] polymer films underwent irreversible electrode processes (Figure 1a–c, dashed lines).

**Figure 2:**
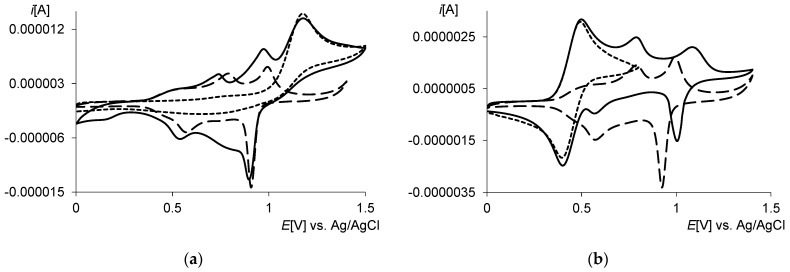
Cyclic voltammograms, 2nd scans, *v* = 0.05 V·s^−1^, vs. Ag/AgCl. (**a**) Pt*poly*[Ni(salcn(Bu))] in TBAH (0.1 mol·dm^−3^)/CH_2_Cl_2_ (after electropolymerization: 3 scans, *v* = 0.05 V·s^−1^)—dashed line; Pt*poly*[Ni(salcn(Bu))] in catechol(10^−3^ mol·dm^−3^)/TBAH/CH_2_Cl_2_—solid line; Pt in catechol/TBAH/CH_2_Cl_2-_dotted line. (**b**) Pt*poly*[Ni(salcn(Bu))] in TBAH (0.1 mol·dm^−3^)/CH_2_Cl_2_ (after electropolymerization: 1 scan, *v* = 0.05 V·s^−1^)—dashed line, Pt*poly*[Ni(salcn(Bu))] in ferrocene (2 × 10^−4^ mol·dm^−3^)/TBAH/CH_2_Cl_2_—solid line; Pt in ferrocene/TBAH/CH_2_Cl_2_—dotted line.

In contrast, the voltammograms of *poly*[Ni(salcn(Bu))] polymers recorded in CH_2_Cl_2_ were so stable that they did not change their characteristics regardless of the electroactive surface coverage (Figure 2, dashed lines and Figure S1,ESI, dashed lines).

They oxidize and reduce in two steps. The kinetics of electrode processes occurring in the films of this polymer is the fastest. *D* values are higher than for *poly*[Ni(salcn)] polymers [38]. The reason is most likely the less compact structure of *poly*[Ni(salcn(Bu))], due to electropolymerization in one direction, forced by substituents in *ortho*- positions and the significant steric effect of *tert*-Bu substituents, facilitating the charge transfer, regardless of the electroactive surface coverage. The weak anode signal at ~0.5 V came from the oxidation of dimers embedded in *poly*[Ni(salcn(Bu))] polymer films, as has been evidenced in previous studies [37].

**Figure 3:**
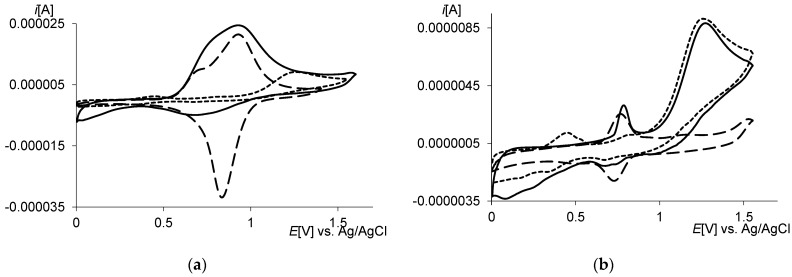
Cyclic voltammograms, 2nd scans, *v* = 0.05 V·s^−1^, vs. Ag/AgCl. Modified electrodes in TBAH (0.1 mol·dm^−3^)/AN (after electropolymerization: 3 scans, *v* = 0.05 V·s^−1^)—dashed lines, modified electrodes in ascorbic acid(10^−3^ mol·dm^−3^)/TBAH/AN—solid lines; Pt in ascorbic acid/TBAH/AN—dotted lines. (**a**) Pt*poly*[Ni(salcn)], (**b**) Pt*poly*[Ni(salcn(Bu))].

In AN, the redox processes in *poly*[Ni(salcn)] films were easier than in CH_2_Cl_2_. The anode peak potentials were lower than in the solutions in CH_2_Cl_2_ (Figure 3a, dashed line, and Figures S2 and S3, ESI), and the electrode processes were less irreversible. For the films with low electroactive surface coverage, even the second step of the anode process and the corresponding reduction process were reversible (Figure S2, ESI). The reason for the easier charge transfer in AN than in CH_2_Cl_2_ is probably the lower viscosity of AN facilitating ion movement and the higher acetonitrile donor number, increasing the association of ions and thus also the ion radius. Larger anions, abandoning the film during cathode processes, leave larger gaps in it, which facilitates the kinetics during anodic processes.

In contrast, the *poly*[Ni(salcn(Bu))] polymer films in AN oxidized only in one step (Figure 3b, dashed line). The reversibility of electrode processes may indicate the influence of the association on the improvement of the quality of charge transfer, but the electrodes modified with films of this polymer are not very stable in the AN medium, and the electropolymerization efficiency is very poor.

**Figure 4:**
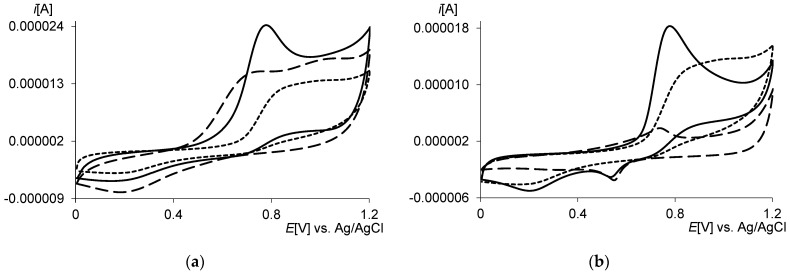
Cyclic voltammograms, 2nd scans, *v* = 0.05 V·s^−1^, vs. Ag/AgCl. Modified electrodes in NaClO_4_/NaOH(0.1 mol·dm^−3^)/H_2_O (after electropolymerization: 3 scans, *v* = 0.05 V·s^−1^)—dashed lines, modified electrodes in NO_2_^−^ (10^−3^ mol·dm^−3^)/NaClO_4_/NaOH/H_2_O—solid lines; Pt in NO_2_^−^/NaClO_4_/NaOH/H_2_O—dotted lines. (**a**) Pt*poly*[Ni(salcn)], (**b**) Pt*poly*[Ni(salcn(Bu))].

In H_2_O, voltammetric curves were recorded in an alkaline environment to prevent hydrolysis of the polymer films. The electrode processes in polymer films in this solvent were more difficult than in CH_2_Cl_2_ and AN. In the case of the *poly*[Ni(salcn)] polymer, the process was a two-step process, but regardless of the electroactive surface coverage, the peaks were less defined, and the processes were more irreversible (Figure 4a, dashed line and Figure S4 ESI) than in the case of the organic solvents used. The *poly*[Ni(salcn(Bu))] polymer oxidized only in one step (Figure 4b, dashed line) and irreversibly.

The analysis of the remaining curves in Figure 1, Figure 2, Figure 3 and Figure 4 (solid and dotted lines) recorded in the solutions of the reducing agent is carried out below in Section 3.2 and Section 3.3.

### 3.2. Electrocatalytic Properties of Modified Electrodes

**Figure 5:**
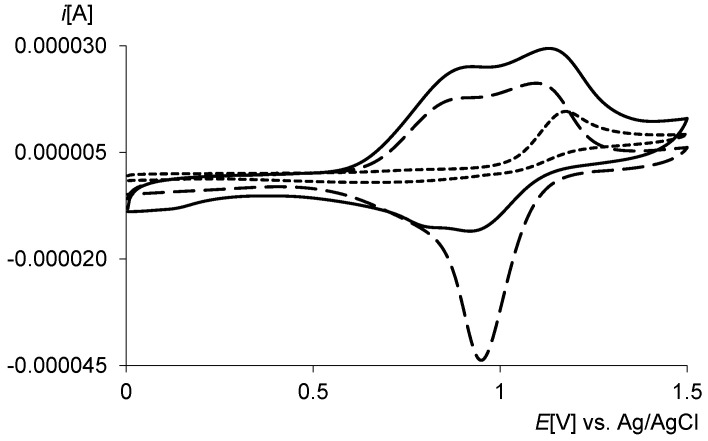
Cyclic voltammograms, 2nd scan, *v* = 0.05 V·s^−1^, vs. Ag/AgCl: Pt*poly*[Ni(salcn)] in TBAH (0.1 mol·dm^−3^)/CH_2_Cl_2_ (after electropolymerization: 3 scans, *v* = 0.05 V·s^−1^)—dashed line, Pt*poly*[Ni(salcn)] in catechol(10^−3^ mol·dm^−3^)/TBAH/CH_2_Cl_2_—solid line; Pt in catechol/TBAH/CH_2_Cl_2_—dotted line.

The electrocatalytic properties of the modified electrodes in CH_2_Cl_2_ were investigated against catechol. Catechol on the unmodified electrode was oxidized at a potential of 1.16 V (Figure 5, dotted line), higher than *poly*[Ni(salcn)] (Figure 5, dashed line), potentially favoring electrocatalysis. The voltammetric curve of the *poly*[Ni(salcn)] polymer film, of average electroactive surface coverage (*Γ* = 8.49 × 10^−9^ mol·cm^−2^), obtained as a result of recording three cycles, at *v* = 0.05 V·s^−1^ in 0.001 mol·dm^−3^ solution of catechol (Figure 5, solid line), showed an increase in the anode peak current and a decrease in the cathode peak current, relative to the peak currents in the absence of a reducer (Figure 5, dashed line), indicating electrocatalytic oxidation of the reducing agent. [43]. However, the electrocatalysis process occurred at a potential higher than the oxidation potential of the *poly*[Ni(salcn)] film, which most probably resulted from the nature of the polymer film. In the investigated film of medium electroactive surface coverage, charge transfer is more difficult than in a film with low electroactive surface coverage [38], and additionally the introduced catechol makes this process even more difficult. This was confirmed by tests carried out in a film with low electroactive surface coverage (Figure S5, solid line, ESI, *Γ* = 4.97 × 10^−9^ mol·cm^−2^), obtained as a result of recording one cycle at *v* = 0.05 V·s^−1^. The voltammograms recorded even in high catechol concentration (0.005 mol·dm^−3^) showed a lower potential for catalysis, as expected for this type of process. Due to the high concentration of the reducing agent in relation to the low concentration of the surface centers of the film with low electroactive surface coverage, apart from electrocatalysis, there was also a process directly on the electrode surface. The higher peak current of the second step of the process recorded during electrocatalysis (Figure S5, solid line, ESI) than that of the catechol peak on the unmodified electrode (Figure S5, dotted line, ESI) was therefore the result of the electrocatalysis effect taking place in the second step and process overlapping directly on the electrode surface. For this reason, the potential of the second step of the process (Figure S5, solid line, ESI) was shifted towards the value corresponding to the second step of polymer oxidation (Figure S5, dashed line, ESI), lower than the oxidation potential of the catechol (Figure S5, dotted line, ESI). The result shows that the *poly*[Ni(salcn)] film with low electroactive surface coverage was involved in catalysis, and at the same time it was not a barrier limiting the movement of catechol to the electrode surface.

The *poly*[Ni(salcn(Bu))] film was prepared in the same way. Using the identical electropolymerization conditions (3 cycles, *v* = 0.05 V·s^−1^), however, a film with less electroactive surface coverage was obtained (Figure 2a, dashed line, *Γ* = 3.41 × 10^−9^ mol·cm^−2^) than the *poly*[Ni(salcn)] film. The *poly*[Ni(salcn(Bu))] films had much less electroactive surface coverage than the *poly*[Ni(salcn)] films obtained in the same way. The reason is the high stabilization of phenoxy radicals by *tert*-Bu substituents [44], which reduces their activity and fast termination during the electrocatalysis process. The consequence of this fact is the presence of dimers and oligomers in *poly*[Ni(salcn(Bu))] films, which has been shown in previous studies [37], which causes the decrease of the surface center densities of the films. The voltammetric curve of the *poly*[Ni(salcn(Bu))] film))] (Figure 2a, dashed line) in 0.001 mol·dm^−3^ catechol solution showed both the electrocatalytic effect and the process taking place directly on the electrode surface (Figure 2a, solid line). There was a clear electrocatalytic effect on the second anode peak—an increase in current and a decrease in the potential of the anode peak (Figure 2a, solid line). However, in the first step of the process, only a shift of the electrocatalysis potential towards lower values was observed. The lower catechol oxidation current directly on the surface of the modified electrode (Figure 2a, solid line) than on the unmodified electrode (Figure 2a, dotted line) resulted from the participation of the polymer in the electrocatalysis of this reducer.

In AN, the electrocatalytic properties of the modified electrodes were investigated against ascorbic acid. Ascorbic acid on the unmodified electrode oxidized at a potential of 1.25 V (Figure 3a, dotted line), higher than the films of the investigated polymers. *Poly*[Ni(salcn)] and *poly*[Ni(salcn(Bu))] polymers films, obtained by the same procedure, in AN showed even greater differences in the concentration of surface centers due to a lower stabilization of *poly*[Ni(salcn(Bu))] in this solvent. The electrocatalysis process was observed in the films of both polymers. Due to the higher concentration of surface centers, in the case of *poly*[Ni(salcn)] (Figure 3a, dashed line, *Γ* = 9.51 × 10^−9^ mol·cm^−2^), only electrocatalysis occurred (Figure 3a, solid line).

The process occurred at higher potential values than those of the *poly*[Ni(salcn)] film, but the difference was lower than that of catechol in a CH_2_Cl_2_ solution. The reason is the easier charge transfer through the film structures in AN. However, in the case of the *poly*[Ni(salcn(Bu))] films with less electroactive surface coverage (Figure 3b, dashed line, *Γ* = 1.85 × 10^−9^ mol·cm^−2^), the process taking place directly on the electrode surface was also observed (Figure 3b, solid line). Its lower oxidation current than on the unmodified electrode (Figure 3b, dotted line) resulted from the participation of polymer in the electrocatalysis of this reducing agent.

**Figure 6:**
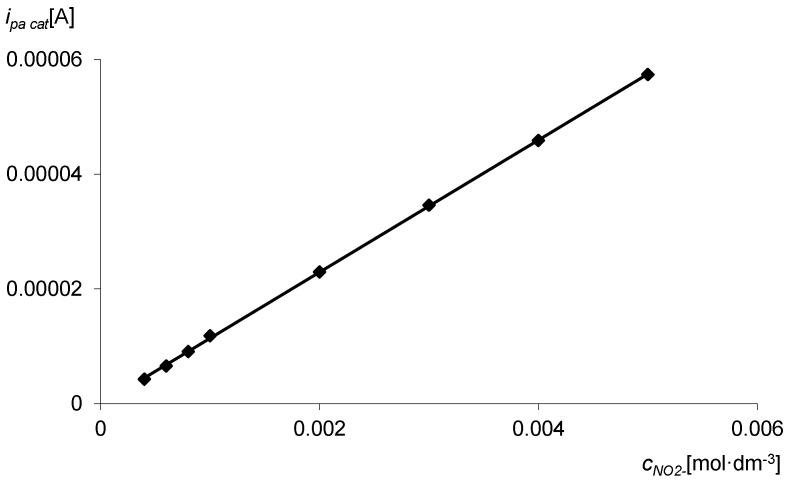
Plots of *i_pa_* NO_2_^−^ catalysis (*i_pa cat_*) vs. NO_2_^−^ concentration at Pt*poly*[Ni(salcn)] (after electropolymerization: 3 scans, *v* = 0.05 V·s^−1^). Data from Figure S6.

In H_2_O, the electrocatalytic properties of the modified electrodes were studied against NO_2_^−^. The nitrate (III) ion on the unmodified electrode was oxidized at a potential of ~0.95 V (Figure 4a, dotted line). The electrocatalysis process was observed in the case of films of both polymers (Figure 4, solid lines). They were obtained by the same procedure. Regardless of the polymer, electrocatalysis led to better shaped peaks and higher peak currents than those recorded on the unmodified electrode (Figure 4, solid lines). This result allows the use of electrodes modified with films of these polymers for the electroanalytical determination of NO_2_^−^ (Figure S6, ESI). This was confirmed by the linear *i_pa cat_* = f(*c_NO2−_*) relationship (Figure 6).

### 3.3. Influence of Electroactive Surface Coverage on Electrocatalysis

#### 3.3.1. Cyclic Voltammetry

Catechol electrocatalysis studies have shown that a film with low electroactive surface coverage is involved in catalysis and enables the process to occur directly on the electrode surface. However, it is difficult to determine whether, for such an electroactive surface coverage, the catalysis process would take place if the catechol oxidation potential was not higher than the polymer oxidation potential. Therefore, ferrocene—a reducing agent with a lower oxidation potential than the catalyst—was selected for the study of the effect of electroactive surface coverage on the result of electrocatalysis. This reducer oxidizes at a potential of 0.49 V (Figure 1c, dotted line). Furthermore, it has a stable electrochemical characteristic, which facilitates research.

In the case of a *poly*[Ni(salcn)] modified electrode, the current response in the ferrocene solution, similarly as in catechol solution depended on the electroactive surface coverage.

For the electrode modified by film with high electroactive surface coverage (Figure 1c, dashed line), in a ferrocene solution with a concentration of 10^−3^ mole·dm^−3^, the electrode process occurred exclusively by electrocatalysis. The current of anode peak for *poly*[Ni(salcn)] increased (Figure 1c, solid line) compared to current without the presence of a reducer (Figure 1c, dashed line), and the cathode peak current disappeared (Figure 1c, solid line). A process related solely to electrocatalysis is indicated by a lack of signals derived from oxidation or reduction of ferrocene directly on the electrode surface (Figure 1c, solid line), which means that the polymer film contained a lot of active centers and was compact enough to not let the reductant particles go through to the electrode surface.

For medium electroactive surface coverage (Figure 1b, dashed line), both the electrocatalytic oxidation of ferrocene and the oxidation directly on the electrode surface were observed (Figure 1b, solid line). Smaller increase in electrocatalysis current than in the case of the modified electrode with high electroactive surface coverage (Figure 1c, solid line) indicated a partial participation of electrocatalysis in the ferrocene oxidation process. The remaining part of the reducer oxidized directly on the surface of the modified electrode, as evidenced by the lower peak current of the ferrocene oxidation on the modified electrode (Figure 1b, solid line) than the peak current at the unmodified electrode (Figure 1b, dotted line). Electrocatalysis was more likely to occur during the first step of catalyst oxidation (Figure 1b, solid line). The anode peak current of this step in the presence of ferrocene increased more than the peak current of the second step of *poly*[Ni(salcn)] oxidation. The reason may be significant differences in ferrocene oxidation and *poly*[Ni(salcn)] in the second step.

**Figure 7:**
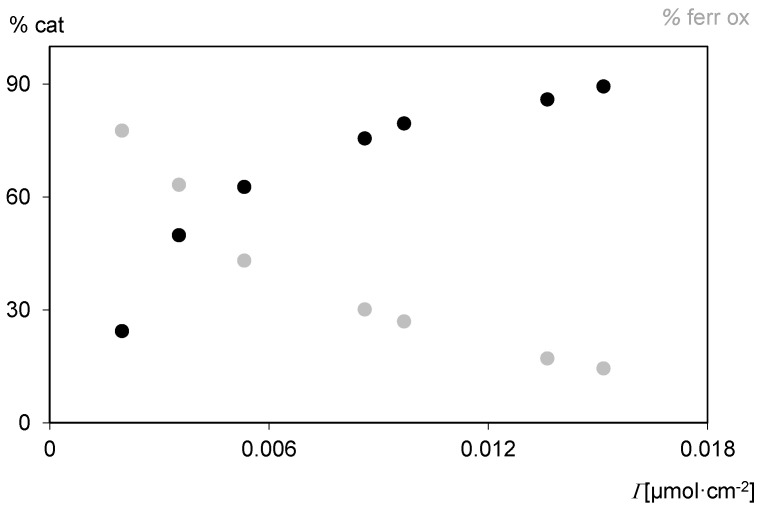
Plots of % catalysis (black points) and % oxidation of ferrocene at Pt*poly*[Ni(salcn)] (% ferr ox) (grey points) vs. electroactive surface coverage (*Γ*). Data from Figure 1 and Figures S7–S12.

The magnitude of the electrocatalytic effect relative to ferrocene as well as the amount of ferrocene oxidation current directly on the surface of the modified electrode depend on the electroactive surface coverage. As their electroactive surface coverage increased, the electrocatalytic effect increased, as indicated by the increasing relationship % cat = f(*Γ*) (Figure 7, black points), where % cat represents the percentage of electrocatalysis on the electrode modified with a polymer film, providing both electrocatalysis and a process directly on the electrode surface (e.g., Figure 1b and Figures S7–S12, ESI, solid lines), compared to an electrode modified with a film assuring only electrocatalysis (Figure 1c, solid line) (% cat = (*i_pa poly_*_[Ni(salcn)] providing catalysis and a process directly on the electrode surface/*ipa poly*[Ni(salcn)] assuring only catalysis)__·100%_). In contrast, the oxidation of ferrocene directly on the surface of the modified electrode decreased with increasing electroactive surface coverage, as evidenced by the decreasing relationship of % ferr ox on Pt*poly* = f(*Γ*) (Figure 7, grey points), where % ferr ox on Pt*poly* means the percentage of oxidation of ferrocene on the electrode surface modified with a polymer film, ensuring both electrocatalysis and the process directly on the electrode surface (e.g., Figure 1b and Figure S7–S12, ESI solid lines), compared to that observed for unmodified electrode (e.g., Figure 1b, dotted line) % ferr ox on Pt*poly* = (*i_pa_*_ferrocene on modified electrode/*ipa* ferrocene on unmodified electrode)__·100%_). Figure 7 thus shows that for each electroactive surface coverage, the size of the electrocatalytic effect and the process taking place directly on the electrode surface complement each other.

For the films with the smallest electroactive surface coverage, the ferrocene oxidation process occurred directly on the electrode surface (Figure 1a, solid line). Ferrocene oxidation peak currents on the surface of the modified electrode (Figure 1a, solid line) and unmodified one (Figure 1a, dotted line) were equal, and the anodic peak currents of *poly*[Ni(salcn)] in the presence of the reducer (Figure 1a, solid line) and its absence (Figure 1a, dashed line) were also equal. Such films are therefore only a barrier, not limiting the diffusion of ferrocene to the electrode surface.

In the case of electrodes modified with *poly*[Ni(salcn(Bu))], no catalytic effects were observed on the voltammetric curves recorded in the presence of ferrocene, regardless of the electroactive surface coverage (Figure 2b and Figure S1, ESI, solid lines). Peak currents of oxidation and reduction of the polymer in the presence (Figure 2b and Figure S1, ESI, solid lines) and absence of ferrocene (Figure 2b and Figure S1, ESI, dashed lines) remained unchanged. In contrast, there were peaks indicating the oxidation and reduction of ferrocene directly on the electrode surface (Figure 2b and Figure S1, ESI, dashed lines), and their currents were analogous to the peak currents recorded on the unmodified electrode (Figure 2b and Figure S1, ESI, dotted lines). This nature of the curves indicates that the *poly*[Ni(salcn(Bu))] polymer films, regardless of their electroactive surface coverage, were only a mechanical barrier for ferrocene, which did not limit the transport of particles through the film in any way. The reason for such properties of *poly*[Ni(salcn(Bu))] films is their structure, resulting from the presence of free dimers and oligomers in films and the presence of *tert*-Bu substituents in the monomers.

#### 3.3.2. EQCM Method

The electrocatalysis process on modified electrodes may take place in several regions: at the interface between the electrode and the polymer film, in the polymer film and at the interface between the polymer film and the solution. The process taking place in the polymer film can occur in its entire volume or only closer to the phase boundary of the electrode–polymer film, or closer to the phase boundary of the polymer film–solution [45].

Voltammetric studies in ferrocene solution showed (Figure 1 and Figures S7–S12, ESI, solid lines) that the ferrocene oxidation process on electrodes modified with *poly*[Ni(salcn)] films can occur directly on the electrode surface under the film layer and/or by electrocatalysis in the polymer film.

EQCM studies allow one to approximate the region in which electrocatalysis occurs, depending on the amount of adsorbed film, by comparing the changes of charges and masses accompanying the anode processes in the electrolyte solution, in the presence and absence of a reducer. Gravimetric curves were obtained on the basis of the Sauerbrey equation [46], showing the relationship between the frequency change (Δf), recorded on the quarc crystal oscillator modified by polymer film, and the change in its mass (Δm). The films with high electroactive surface coverage deposited on the surface of a quartz resonator are stiff, constituent-constant and are obtained by Faraday processes, as indicated by the linearity of the Δ*m = f*(*Q*) relationship [30,47] (Figure S13, ESI). Slight deviations from linearity in the range of the highest mass change values observed in the films with less electroactive surface coverage (Figure S14, ESI) may indicate failure to meet one or more of the listed characteristics at higher potentials. The linearity of the obtained dependencies, in the predominant range of mass changes, allows one to link directly the frequency recorded on the quarc crystal oscillator modified with *poly*[Ni(salcn)] polymer films with the mass transported into these films.

**Figure 8:**
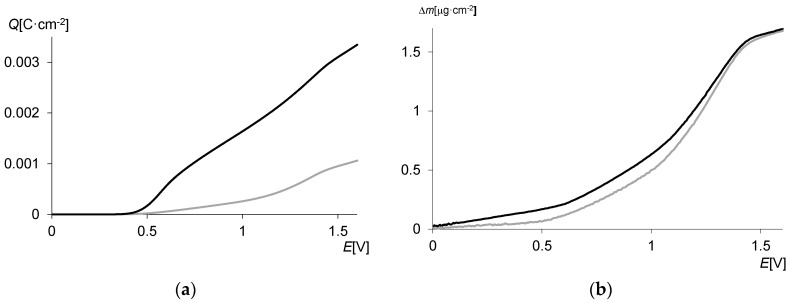
Oxidation processes of *poly*[Ni(salcn)] (after electropolymerization: 3 scans, *v* = 0.5 V·s^−1^) at modified Pt/quartz crystal, *v* = 0.05 V·s^−1^, vs. Ag/AgCl; in TBAH (0.1 mol·dm^−3^)/CH_2_Cl_2_—grey lines, in ferrocene (10^−3^ mol·dm^−3^)/TBAH/CH_2_Cl_2_—black lines; (**a**) plots *Q* vs. *E*, (**b**) plots Δ*m* vs. *E*.

The research was carried out for modified electrodes, obtained as a result of three electropolymerization cycles, at different polarization rates, which allowed us to obtain a sufficiently wide spectrum of the amount of adsorbed film. The charge and mass change were read at the potential corresponding to the end of the anode process taking place in the polymer (approx. 1.6 V, depending on the amount of adsorbed film), on the basis of *Q = f*(*E*) and Δ*m = f*(*E*) curves (Figure 8 and Figures S15–S21, ESI). The charge values obtained in the ferrocene solution determined the total charge that flowed during the oxidation of ferrocene directly on the electrode surface and by electrocatalysis.

As the amount of adsorbed film (the polarization rate of the working electrode) increased, and the charge that flowed during the anode process (Table 1), recorded both in the absence (*Q_el_*) and in the presence of ferrocene (*Q_el–fer_*), increased, which resulted from the increasing number of active centers of polymer films. On the other hand, the *Q_el–fer_/Q_el_* ratio (Table 1) decreased and indicated that the share of ferrocene oxidation in the entire anodic process increased in films with increasing amounts of electroactive surface coverage, but to a lesser extent. Voltammetric studies have shown that with the increase in amount of adsorbed film, the oxidation of ferrocene directly on the electrode surface decreases, and the oxidation by electrocatalysis increases. Thus, the decreasing *Q_el–fer_/Q_el_* relationship indicates that the reduction in the share of ferrocene oxidation taking place directly on the electrode surface is not fully compensated by the increase in the electrocatalytic oxidation of the reducer. This effect is most likely due to kinetic constraints that progress with increasing amount of adsorbed film [38].

The next parameter, Δ*m* (Table 1), increased with the increase in the amount of adsorbed film during the anode process, both recorded in the absence (Δ*m_el_*) as well as in the presence of ferrocene (Δ*m_el–fer_*), which, like in the case of charge changes, resulted from the increasing number of active centers of polymer films. On the other hand, the Δ*m_el–fer_*/Δ*m_el_* (Table 1) changed, but irregularly. For the film with the smallest electroactive surface coverage, it was 1, indicating no disturbance in mass transport in the presence of ferrocene. As the amount of adsorbed film increased, the Δ*m_el–fer_*/Δ*m_el_* value increased to a certain level, then decreased and returned to 1 for the film with the highest electroactive surface coverage. The increase in the Δ*m_el–fer_*/Δ*m_el_* ratio with the increase in the amount of adsorbed film is the result of the increasing share of electrocatalysis in the entire anode process. On the other hand, successive, ever lower values of Δ*m_el–fer_*/Δ*m_el_* for films with increasing amount of electroactive surface coverage may result from the limitation of electrocatalysis or may indicate that only the electrocatalysis inside the polymer film is limited. However, the results of voltammetric studies exclude the limitation of total electrocatalysis. The share of this process increased with increasing the amount of adsorbed film (Figure 6). It can therefore be concluded that in films with increasing amount of electroactive surface coverage only the electrocatalysis within the film is limited. This was confirmed by the value of Δ*m_el–fer_*/Δ*m_el_* equal to 1 (Table 1) for the films with the highest electroactive surface coverage, indicating electrocatalysis only at the polymer–electrolyte interface. The limitations of electrocatalysis inside the film may also be confirmed by the decreasing *Q_el–fer_/Q_el_* relationship, indicating that the reduction in the share of ferrocene oxidation directly on the electrode surface is not fully compensated by the increase in electrocatalysis. Limiting the electrocatalysis inside the films, along with the increase of the amount of adsorbed film, is justified by decreasing *cD^1/2^* values in this direction [38].

#### 3.3.3. RDE Method

Another method that characterizes the oxidation process of the reducing agent on modified electrodes is the RDE method.

**Figure 9:**
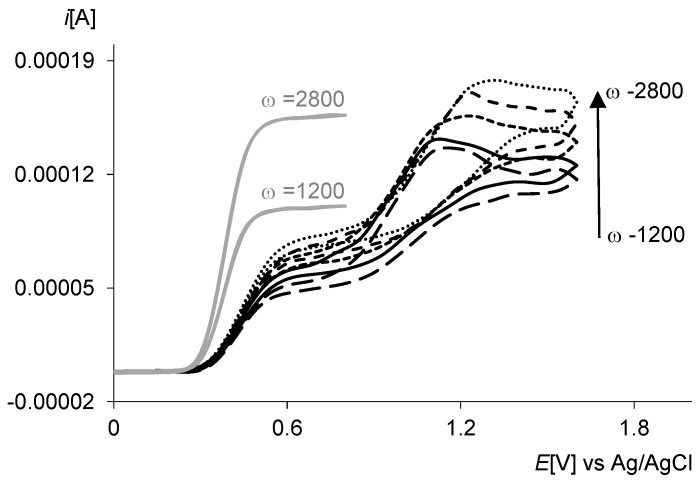
Cyclic voltammograms, 2nd scan, *v* = 0.05 V·s^−1^, vs. Ag/AgCl, *ω*: 1200, 1600, 2000, 2400, 2800 rad^1/2^·s^−1/2^; at rotating Pt disc electrode in ferrocene (10^−3^ mol·dm^−3^)//TBAH/CH_2_Cl_2_—grey lines; at rotating Pt*poly*[Ni(salcn)] disc electrode (*Γ* = 5.39 × 10^−9^ mol·cm^−2^) in ferroceneTBAH (0.1 mol·dm^−3^)/CH_2_Cl_2_—black lines.

The voltammetric curves of the redox processes obtained for ferrocene (10^−3^ mol·dm^3^) on the unmodified electrode showed a plateau at a potential of 0.48 V, depending on the rotation rate of the electrode (*ω*) (Figure 9, grey lines), according to the Levich equation [43].

Voltammograms for redox processes recorded for ferrocene on electrodes modified with medium electroactive surface coverage were characterized by a plateau at ~0.55 V, derived from the oxidation and reduction of ferrocene directly on the surface of the modified electrode and peaks at ~1.1 V (*ω* = 1200 [rad^1/2^·s^−1/2^]), derived from the electrocatalytic oxidation of ferrocene by the polymer (Figure 9, black lines and Figures S22 and S23, ESI). As the electrode rotation rate increased, the peak potentials shifted towards higher values, which resulted from the irreversibility of the electrode process. In the films with low electroactive surface coverage (Figure S24, ESI), electrode processes occurred more easily. They oxidized in two steps, and the first step occurred already at a potential of ~0.8 V. The participation of electrocatalysis in the ferrocene oxidation process was indicated by an increase in peak currents along with an increase in the electrode rotation rate. In the supporting electrolyte solution, in the absence of ferrocene, the currents of the polymer oxidation peaks were essentially independent of the rotation rate (Figure S25, ESI).

In contrast, with films with high electroactive surface coverage, no plateaus were observed at ~0.55 V (Figure S26, ESI), indicating that the ferrocene was not oxidized directly at the electrode surface. There was also no increase in peak currents with increasing electrode rotation rate, which may indicate that no electrocatalysis occurred at all or only occurs at the polymer film–solution interface. Voltammetric studies on nonrotating modified electrode exclude the first possibility. Therefore, it can be concluded that such a character of the curves is most likely a consequence of a film with too high electroactive surface coverage, preventing the movement of molecules through its structure. The presence of peaks instead of plateaus does not allow for a broader characterization of the electrocatalysis process. However, the % cat = f(*Γ*) relationship (Figure 7, black points), which increases with increasing an electroactive surface coverage to a lesser extent, and the results of EQCM studies presented above, may support the hypothesis that in films with high electroactive surface coverage, electrocatalysis occurs only at the interface of the polymer film–solution interface.

**Figure 10:**
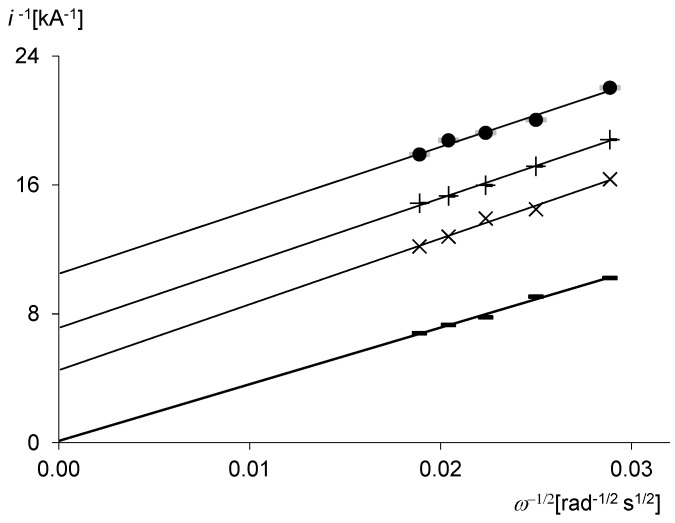
Koutecky–Levich plots for ferrocene oxidation processes at rotating Pt*poly*[Ni(salcn)] disc electrode; (•)—*Γ* = 12.7 × 10^−9^, (+)—*Γ* = 8.51 × 10^−9^, (x)—*Γ* = 5.39 × 10^−9^, (–)—*Γ* = 1.22 × 10^−9^ mol·cm^−2^. Data from Figure 9 and Figures S22–S24.

However, in the case of electrodes modified with low and medium electroactive surface coverage (Figure 9, black lines and Figures S22–S24, ESI), the process of ferrocene oxidation directly on the surface of the modified electrode, marked as plateau, allowed for the analysis of this step of the process. The limiting current on the modified electrodes (Figure 9, black lines and Figures S22–S24, ESI) was lower than on the unmodified ones (Figure 9, grey lines) and indicated a different charge transport rate in the film and in the solution [43]. The Koutecky–Levich equation [43] allowed us to distinguish these effects. The *i_l_*^−1^ = f(*ω*^−1/2^) (K–L) relationships for ferrocene oxidation processes showed a linear character, with the intersection points above 0 (Figure 10) and thus their *membrane model* nature [43]. The limiting current corresponded to the oxidation of ferrocene directly on the electrode surface. Therefore, the rate of the reaction may be controlled by the rate of electron transfer at the interface between the electrode surface and polymer or the rate of mass transport through the film [9,45]. The solution is provided by the analysis of *k’* values (effective heterogeneous rate constants for the modified electrodes), depending on the concentrations of the active surface centers of the films (*Γ*). The values of *k’*, determined on the basis of the points of intersection of the *i_l_*^−1^ = f(*ω*^−1/2^) function, decreased with increasing the electroactive surface coverage (Table 2) and thus indicated a process controlled by mass transport through the film [45].

## 4. Conclusions

The *poly*[Ni(salcn(Bu))] polymer films have less electroactive surface coverage than the *poly*[Ni(salcn)] films obtained by the same procedure. The reason is the high stabilization of phenoxy radicals by *tert*-Bu substituents, which reduces their activity and fast termination during the electropolymerization process. Due to differences in the electroactive surface coverage, the results of the electrode processes taking place in the reducing solutions are different. They were investigated by cyclic voltammetry, EQCM and RDE methods.

In reducing agent solutions, oxidizing at higher potentials (catechol, ascorbic acid and nitrate (III) ions) than the polymer oxidation potentials, electrocatalysis is observed on electrodes modified with films of both polymers. For *poly*[Ni(salcn)] films with more electroactive surface coverage, this is only electrocatalysis. For films of this polymer with less electroactive surface coverage, the process taking place directly on the electrode surface is also observed. On the other hand, in the case of *poly*[Ni(salcn(Bu))] films, regardless of their electroactive surface coverage, the oxidation of the reducers occurs by electrocatalysis and directly on the electrode surface.

Electrocatalysis leads to higher and, in the case of NO_2_^−^, better defined peaks, which allows for the use of electrodes modified with the investigated polymers for the electroanalytical determination of nitrate (III) ions.

The analysis of the influence of the electroactive surface coverage on the nature of the oxidation of the reducing agent was carried out in reductant solutions, oxidizing at a lower potential value (ferrocene) than the oxidation potential of the polymers.

The *poly*[Ni(salcn(Bu))] polymer films, regardless of their electroactive surface coverage, are only a mechanical barrier to ferrocene, not limiting the transport of molecules through the film. This is most likely due to the less compact structure of *poly*[Ni(salcn(Bu))], due to electropolymerization in one active direction forced by the substituents in the *ortho*- positions and the significant steric effect of the *tert*-Bu substituents.

On the other hand, *poly*[Ni(salcn)] films react in a manner dependent on their electroactive surface coverage. The films with high electroactive surface coverage ensure the exclusive electrocatalysis of ferrocene, while the films with low electroactive surface coverage—only the process on the electrode surface. The films of intermediate electroactive surface coverage allow both electrocatalysis and a process directly on the electrode surface. As their electroactive surface coverage increases, the electrocatalytic effect increases, and the process taking place directly on the electrode surface decreases, as indicated by the relationships % cat = f(*Γ*) and % ox ferr on Pt*poly* = f(*Γ*).

EQCM studies have brought closer the region in which electrocatalysis occurs by comparing the changes of charges and masses accompanying the anode processes in the electrolyte solution, in the presence (*Q_el–fer_* and Δ*m_el–fer_*) and in the absence of ferrocene (*Q_el_* and Δ*m_el_*), on the basis of the *Q = f*(*E*) and Δ*m = f*(*E*). The *Q_el_* and *Q_el–fer_* fer values increased with the increase of the amount of adsorbed film, and the decreasing *Q_el–fer_/Q_el_* relationship indicates a slower and slower progress of ferrocene oxidation. In this process, the share of ferrocene oxidation, taking place directly on the electrode surface, decreases and is not fully compensated by the increase in electrocatalytic oxidation of the reducer. This effect is most likely due to kinetic constraints that progress with increasing the amount of adsorbed film. Moreover, the increasing Δ*m_el_* and Δ*m_el–fer_* values and the irregularly changing Δ*m_el–fer_*/Δ*m_el_* relationship, along with the increase in the amount of adsorbed film (from 1, through growth and reduction to 1) indicate the limitations of electrocatalysis inside the films with an increasing amount of electroactive surface coverage and of electrocatalysis only at the polymer–electrolyte interface in the films with the highest electroactive surface coverage.

The electrocatalysis occurring only at the polymer–electrolyte interface in the films with high electroactive surface coverage is confirmed by RDE studies, due to the lack of an increase in polymer peak currents with an increase in the electrode rotation rate. In the films with less electroactive surface coverage, apart from electrocatalysis, the oxidation of ferrocene is observed directly on the electrode surface in the form of a plateau, taking place according to the *membrane model*. The *i_l_*^−1^ = f(*ω*^−1/2^) relationships on the basis of the Koutecky–Levich equation for the oxidation of ferrocene show a linear character, with intersection points above 0. This process is controlled by mass transport through the film, which is indicated by decreasing values of *k’* (effective heterogeneous rate constants for the modified electrodes) with increases in the electroactive surface coverage.

## Figures and Tables

**Table 1:** Data from EQCM method for oxidation of *poly*[Ni(salcn)], obtained as a result of 3 electropolymerization scans at various scan rates, in 10^−1^ mol·dm^−3^ TBAH/CH_2_Cl_2_ in the presence and absence of ferrocene (*c* = 10^−3^ mol·cm^3^).

*V* (V·s^−1^)	*Q_el_* (C·cm^2^)	*Q_el–fer_* (C·cm^2^)	*Q_el–fer_/Q_el_*	Δ*m_el_* (μg·cm^2^)	Δ*m_el–fer_* (μg·cm^2^)	Δ*m_el–fer_/* Δ*m_el_*
0.50	0.0010	0.0036	3.20	1.66	1.67	1.00
0.40.	0.0015	0.0046	2.97	1.79	1.96	1.09
0.30	0.0017	0.0048	2.91	2.28	2.62	1.15
0.20	0.0020	0.0054	2.67	2.76	3.71	1.34
0.05.	0.0046	0.0097	2.13	5.61	6.82	1.22
0.04	0.0058	0.0107	1.84	6.35	7.52	1.18
0.02	0.0087	0.0139	1.60	10.9	12.0	1.10
0.01	0.0170	0.0260	1.49	24.0	24.0	1.00

*Q_el,_Q_el–fer_*—total charge flown in the absence and presence of ferrocene, respectively; Δ*m_el_*, Δ*m_el–fer_*—the change in film mass as occurred in the absence and presence of the ferrocene, respectively.

**Table 2:** Values of effective heterogeneous rate constants for the Pt*poly*[Ni(salcn)] (*k’*) in 10^−1^ mol·dm^−3^ TBAH/CH_2_Cl_2_/10^−3^ mol·dm^−3^ ferrocene.

*Γ* (mol cm^−2^)	*k’* (cm s^−1^)
1.22 × 10^−9^	0.43
5.39 × 10^−9^	0.011
8.51 × 10^−9^	0.0068
12.7 × 10^−9^	0.0046

## Data Availability

Data are contained within the article.

## Supplementary Materials

The following are available online at https://www.mdpi.com/article/10.3390/ma15010191/s1, Figure S1: Cyclic voltammograms, 2nd scan, *v* = 0.05 V·s^−1^, vs. Ag/AgCl: Pt*poly*[Ni(salcn(Bu))], Figure S2: Cyclic voltammogram at Pt*poly*[Ni(salcn)] (after electropolymerization: 1 scan, *v* = 0.05 V·s^−1^) in TBAH (0.1 mol·dm^−3^)/AN, Figure S3: Cyclic voltammogram at Pt*poly*[Ni(salcn)] (after electropolymerization: 10 scans, *v* = 0.05 V·s^−1^) in TBAH (0.1 mol·dm^−3^)/AN, Figure S4: Cyclic voltammogram at Pt*poly*[Ni(salcn)] (after electropolymerization: 10 scans, *v* = 0.05 V·s^−1^) in NaClO_4_/NaOH(0.1 mol·dm^−3^)/H_2_O, Figure S5: Cyclic voltammograms, 2nd scan, *v* = 0.05 V·s^−1^, vs. Ag/AgCl: Pt*poly*[Ni(salcn)], Figure S6: Cyclic voltammograms at Pt*poly*[Ni(salcn)] (after electropolymerization: 3 scans, *v* = 0.05 V·s^−1^) in NO_2_^−^(4 × 10^−4^–5 × 10^−3^ mol·dm^−3^)/ NaClO_4_/ NaOH(0.1 mol·dm^−3^)/ H_2_O, Figure S7: Cyclic voltammograms, 2nd scan, *v* = 0.05 V·s^−1^, vs. Ag/AgCl: Pt*poly*[Ni(salcn)] (*Γ* = 1.52·10^−8^ mol·cm^−2^), Figure S8: Cyclic voltammograms, 2nd scan, *v* = 0.05 V·s^−1^, vs. Ag/AgCl: Pt*poly*[Ni(salcn)] (*Γ* = 1.36·10^−8^ mol·cm^−2^), Figure S9: Cyclic voltammograms, 2nd scan, *v* = 0.05 V·s^−1^, vs. Ag/AgCl: Pt*poly*[Ni(salcn)] (*Γ* = 9.69 × 10^−9^ mol·cm^−2^), Figure S10: Cyclic voltammograms, 2nd scan, *v* = 0.05 V·s^−1^, vs. Ag/AgCl: Pt*poly*[Ni(salcn)] (*Γ* = 5.32·10^−9^ mol·cm^−2^), Figure S11: Cyclic voltammograms, 2nd scan, *v* = 0.05 V·s^−1^, vs. Ag/AgCl: Pt*poly*[Ni(salcn)] (*Γ* = 3.54 × 10^−9^ mol·cm^−2^), Figure S12: Cyclic voltammograms, 2nd scan, *v* = 0.05 V·s^−1^, vs. Ag/AgCl: Pt*poly*[Ni(salcn)] (*Γ* = 1.98 × 10^−9^ mol·cm^−2^), Figure S13: Δ*m* vs. *Q* plot for oxidation process of *poly*[Ni(salcn)] (after electropolymerization: 3 scans, *v* = 0.01 V·s^−1^), Figure S14: Δ*m* vs. *Q* plot for oxidation process of *poly*[Ni(salcn)] (after electropolymerization: 3 scans, *v* = 0.5 V·s^−1^), Figure S15: Oxidation process of *poly*[Ni(salcn)] (after electropolymerization: 3 scans, *v* = 0.4 V·s^−1^) at modified Pt/quartz crystal, Figure S16: Oxidation process of *poly*[Ni(salcn)] (after electropolymerization: 3 scans, *v* = 0.3 V·s^−1^) at modified Pt/quartz crystal, Figure S17: Oxidation process of *poly*[Ni(salcn)] (after electropolymerization: 3 scans, *v* = 0.2 V·s^−1^) at modified Pt/quartz crystal, Figure S18: Oxidation process of *poly*[Ni(salcn)] (after electropolymerization: 3 scans, *v* = 0.05 V·s^−1^) at modified Pt/quartz crystal, Figure S19: Oxidation process of *poly*[Ni(salcn)] (after electropolymerization: 3 scans, *v* = 0.04 V·s^−1^) at modified Pt/quartz crystal, Figure S20: Oxidation process of *poly*[Ni(salcn)] (after electropolymerization: 3 scans, *v* = 0.02 V·s^−1^) at modified Pt/quartz crystal, Figure S21: Oxidation process of *poly*[Ni(salcn)] (after electropolymerization: 3 scans, *v* = 0.01 V·s^−1^) at modified Pt/quartz crystal, Figure S22: Cyclic voltammograms at rotating Pt*poly*[Ni(salcn)] disc electrode (*Γ* = 8.51 × 10^−9^ mol·cm^−2^), Figure S23: Cyclic voltammograms at rotating Pt*poly*[Ni(salcn)] disc electrode (*Γ* = 12.7 × 10^−9^ mol·cm^−2^), Figure S24: Cyclic voltammograms at rotating Pt*poly*[Ni(salcn)] disc electrode (*Γ* = 1.22 × 10^−9^ mol·cm^−2^), Figure S25: Cyclic voltammograms at rotating Pt*poly*[Ni(salcn)] disc electrode (*Γ* = 6.27 × 10^−9^ mol·cm^−2^), Figure S26: Cyclic voltammograms at rotating Pt*poly*[Ni(salcn)] disc electrode (*Γ* = 17.1 × 10^−9^ mol·cm^−2^).
