# Supplementary material for: Electrocatalytic Properties of Ni(II) Schiff Base Complex Polymer Films
Source: Materials (Basel). 2021 Dec 28;15(1):191. doi: 10.3390/ma15010191 (PMC8745840; doi:10.3390/ma15010191)
Supplement: Supplementary file 1 [file materials-15-00191-s001.zip › materials-1503619-supplementary.pdf]

Supplementary materials

Electrocatalytic Properties of Ni(II) Schiff Base Complex Polymer Films

Danuta Tomczyk [1,*], Wiktor Bukowski [2], Karol Bester [2] and Michalina Kaczmarek [1]

[1] Department of Inorganic and Analytical Chemistry, University of Łódź, ul. Tamka 12, 91-403 Łódź, Poland; michalina.kaczmarek@unilodz.eu

[2] Faculty of Chemistry, Rzeszów University of Technology, Al. Powstańców W-wy 6, 35-959 Rzeszów, Poland; wbuk@sd.prz.edu.pl (W.B.); bester_k@prz.edu.pl (K.B.)

* Correspondence: danuta.tomczyk@chemia.uni.lodz.pl

Figures

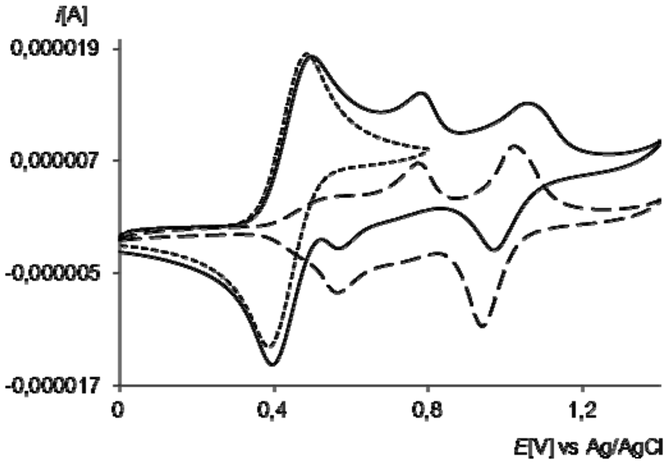

Figure S1. Cyclic voltammograms, 2nd scan, v = 0.05 V·s^{-1}, *vs.* Ag/AgCl: Pt*poly*[Ni(salcn(Bu))] in TBAH (0.1 mol·dm^{-3})/CH_2Cl_2 (after electropolymerization: 20 scans, v = 0.05 V·s^{-1})—dashed line, Pt*poly*[Ni(salcn)] in ferrocene(10^{-3} mol·dm^{-3})/TBAH/CH_2Cl_2—solid line; Pt in ferrocene/TBAH/CH_2Cl_2—dotted line.

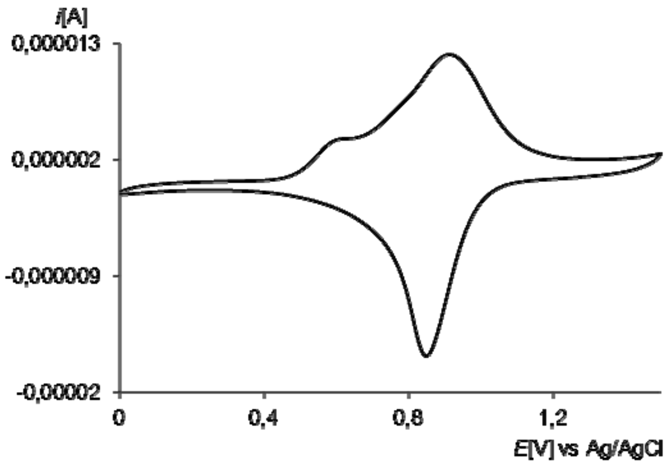

Figure S2. Cyclic voltammogram at Pt*poly*[Ni(salcn)] (after electropolymerization: 1 scan, v = 0.05 $V \cdot s^{-1}$) in TBAH(0.1 $mol \cdot dm^{-3}$)/AN, 2nd scan, v = 0.05 $V \cdot s^{-1}$, *vs.* Ag/AgCl.

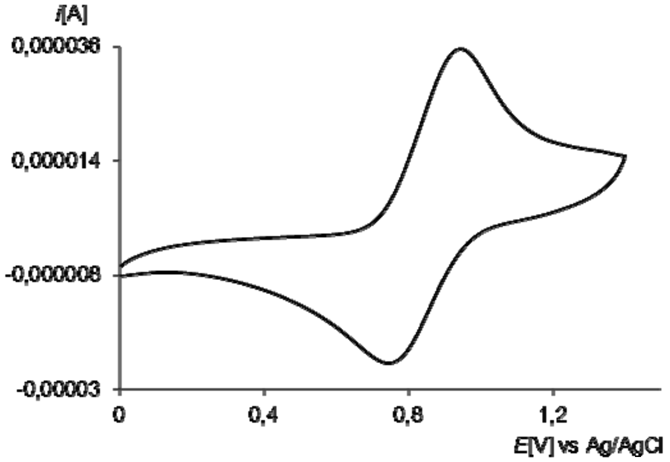

\

Figure S3. Cyclic voltammogram at Pt*poly*[Ni(salcn)] (after electropolymerization: 10 scans, v = 0.05 $V \cdot s^{-1}$) in TBAH(0.1 $mol \cdot dm^{-3}$)/AN, 2nd scan, v = 0.05 $V \cdot s^{-1}$, *vs.* Ag/AgCl.

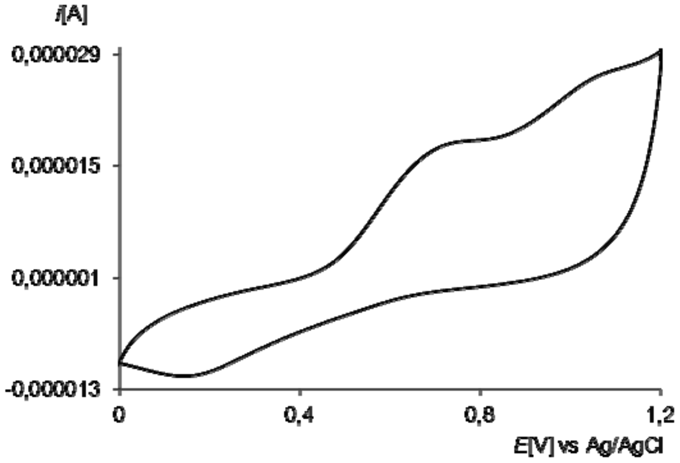

Figure S4. Cyclic voltammogram at Pt*poly*[Ni(salcn)] (after electropolymerization: 10 scans, v = 0.05 $V \cdot s^{-1}$) in $NaClO_4$/NaOH(0.1 $mol \cdot dm^{-3}$)/H_2O, 2nd scan, v = 0.05 $V \cdot s^{-1}$, *vs.* Ag/AgCl.

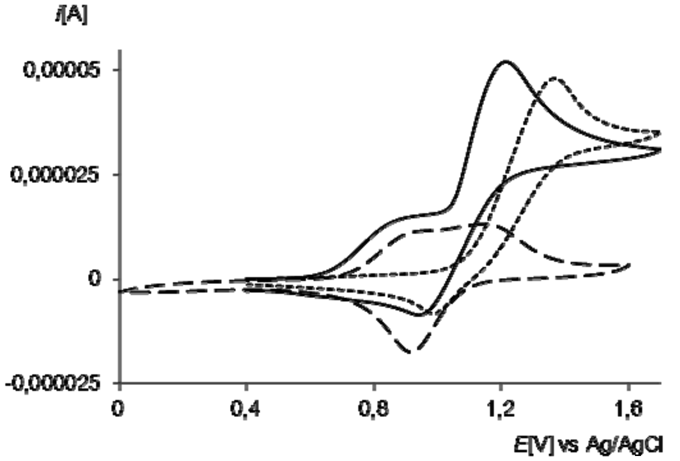

Figure S5. Cyclic voltammograms, 2nd scan, v = 0.05 V·s^{-1}, *vs.* Ag/AgCl: Pt*poly*[Ni(salcn)] in TBAH(0.1 mol·dm^{-3})/CH_2Cl_2 (after electropolymerization: 1 scan, v = 0.05 V·s^{-1})—dashed line, Pt*poly*[Ni(salcn)] in catechol(5 × 10^{-3} mol·dm^{-3})/TBAH/CH_2Cl_2—solid line; Pt in catechol/TBAH/CH_2Cl_2—dotted line.

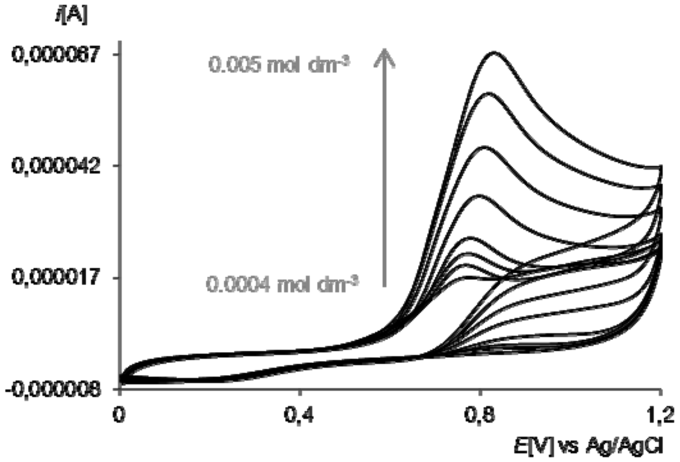

Figure S6. Cyclic voltammograms at Pt*poly*[Ni(salcn)] (after electropolymerization: 3 scans, v = 0.05 V·s^{-1}) in NO_2^- (4 × 10^{-4}–5 × 10^{-3} mol·dm^{-3})/$NaClO_4$/NaOH(0.1 mol·dm^{-3})/H_2O, 2nd scan, v = 0.05 V·s^{-1}, *vs.* Ag/AgCl.

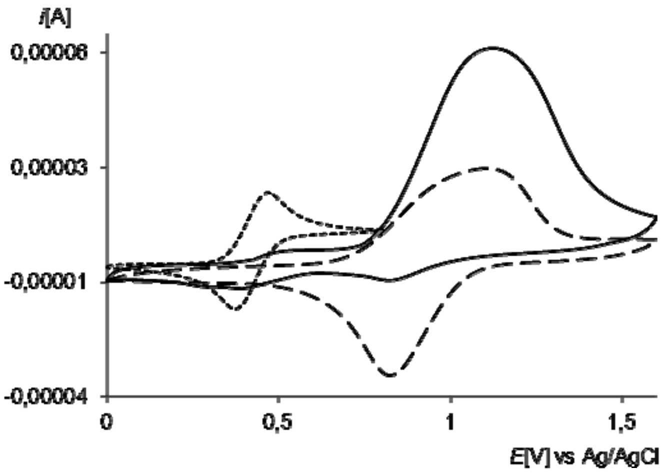

Figure S7. Cyclic voltammograms, 2nd scan, v = 0.05 V·s^{-1}, *vs.* Ag/AgCl: Pt*poly*[Ni(salcn)] (Γ= 1.52 × 10^{-8} mol·cm^{-2}) in TBAH(0.1 mol·dm^{-3})/CH_2Cl_2—dashed line, Pt*poly*[Ni(salcn)] in ferrocene(10^{-3} mol·dm^{-3})/TBAH/CH_2Cl_2—solid line; Pt in ferrocene/TBAH/CH_2Cl_2—dotted line.

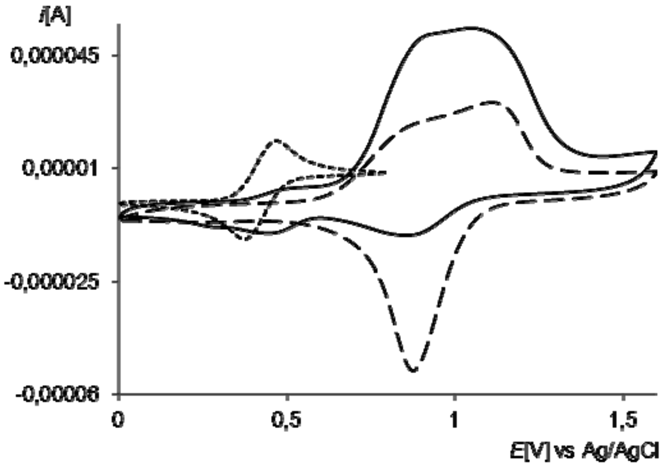

Figure S8. Cyclic voltammograms, 2nd scan, v = 0.05 V·s^{-1}, *vs.* Ag/AgCl: Pt*poly*[Ni(salcn)] (Γ= 1.36 × 10^{-8} mol·cm^{-2}) in TBAH(0.1 mol·dm^{-3})/CH_2Cl_2—dashed line, Pt*poly*[Ni(salcn)] in ferrocene(10^{-3} mol·dm^{-3})/TBAH/CH_2Cl_2—solid line; Pt in ferrocene/TBAH/CH_2Cl_2—dotted line.

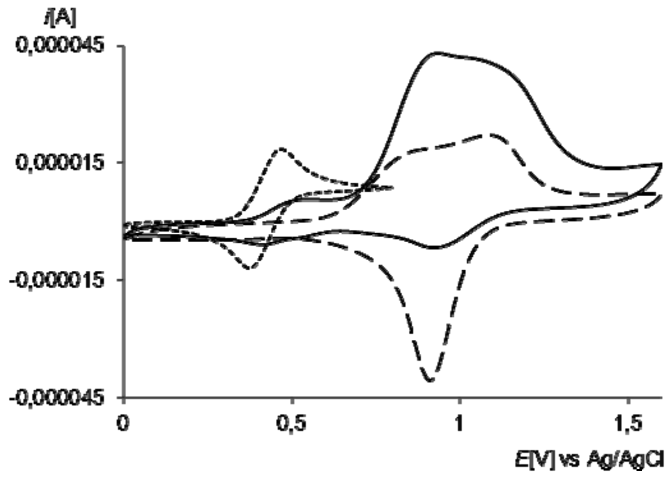

Figure S9. Cyclic voltammograms, 2nd scan, v = 0.05 V·s^{-1}, *vs.* Ag/AgCl: Pt*poly*[Ni(salcn)] (Γ = 9.69 × 10^{-9} mol·cm^{-2}) in TBAH(0.1 mol·dm^{-3})/CH_2Cl_2—dashed line, Pt*poly*[Ni(salcn)] in ferrocene(10^{-3} mol·dm^{-3})/TBAH/CH_2Cl_2—solid line; Pt in ferrocene/TBAH/CH_2Cl_2—dotted line.

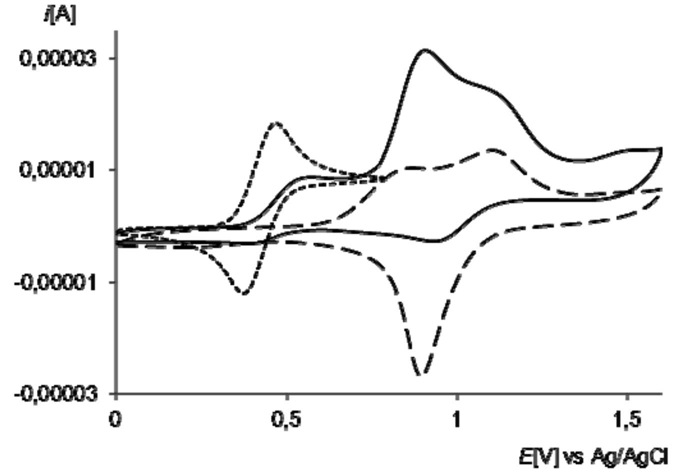

Figure S10. Cyclic voltammograms, 2nd scan, v = 0.05 V·s^{-1}, *vs.* Ag/AgCl: Pt*poly*[Ni(salcn)] (Γ = 5.32 × 10^{-9} mol·cm^{-2}) in TBAH(0.1 mol·dm^{-3})/CH_2Cl_2—dashed line, Pt*poly*[Ni(salcn)] in ferrocene(10^{-3} mol·dm^{-3})/TBAH/CH_2Cl_2—solid line; Pt in ferrocene/TBAH/CH_2Cl_2—dotted line.

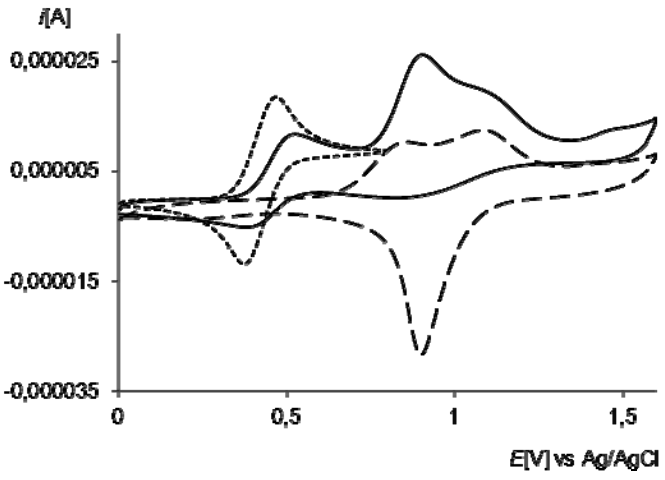

Figure S11. Cyclic voltammograms, 2nd scan, v = 0.05 V·s^{-1}, *vs.* Ag/AgCl: Pt*poly*[Ni(salcn)] (Γ = 3.54 × 10^{-9} mol·cm^{-2}) in TBAH(0.1 mol·dm^{-3})/CH_2Cl_2—dashed line, Pt*poly*[Ni(salcn)] in ferrocene(10^{-3} mol·dm^{-3})/TBAH/CH_2Cl_2—solid line; Pt in ferrocene/TBAH/CH_2Cl_2—dotted line.

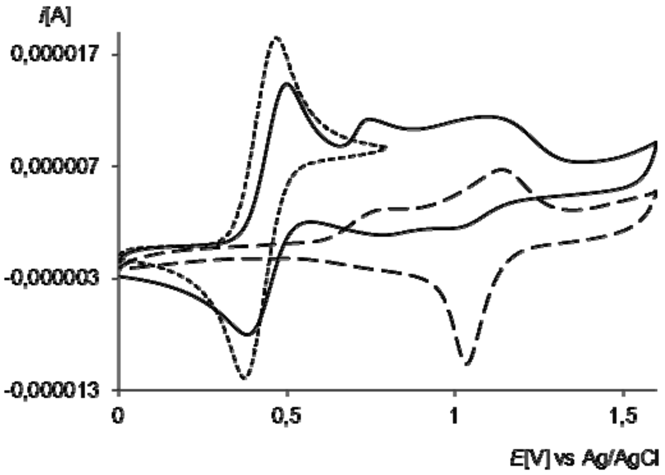

Figure S12. Cyclic voltammograms, 2nd scan, v = 0.05 V·s^{-1}, *vs.* Ag/AgCl: Pt*poly*[Ni(salcn)] (Γ = 1.98 × 10^{-9} mol·cm^{-2}) in TBAH(0.1 mol·dm^{-3})/CH_2Cl_2—dashed line, Pt*poly*[Ni(salcn)] in ferrocene(10^{-3} mol·dm^{-3})/TBAH/CH_2Cl_2—solid line; Pt in ferrocene/TBAH/CH_2Cl_2—dotted line.

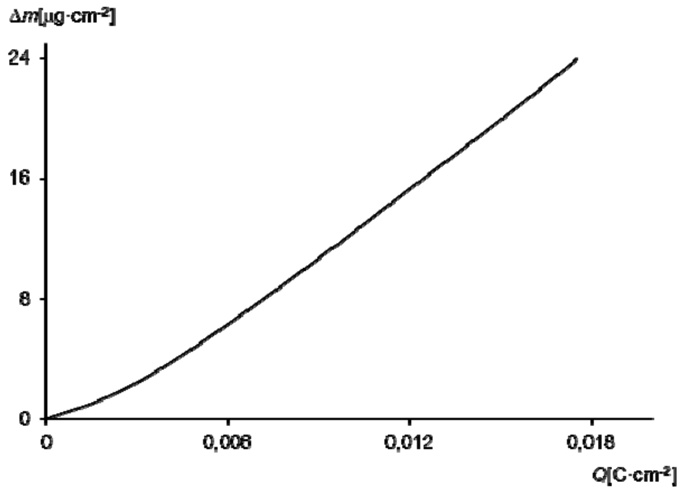

Figure S13. Δm vs Q plot for oxidation process of *poly*[Ni(salcn)] (after electropolymerization: 3 scans, v = 0.01 $V{\cdot}s^{-1}$) in TBAH(0.1 $mol{\cdot}dm^{-3}$)/CH_2Cl_2 at modified Pt/quartz crystal, v = 0.05 $V{\cdot}s^{-1}$, vs. Ag/AgCl.

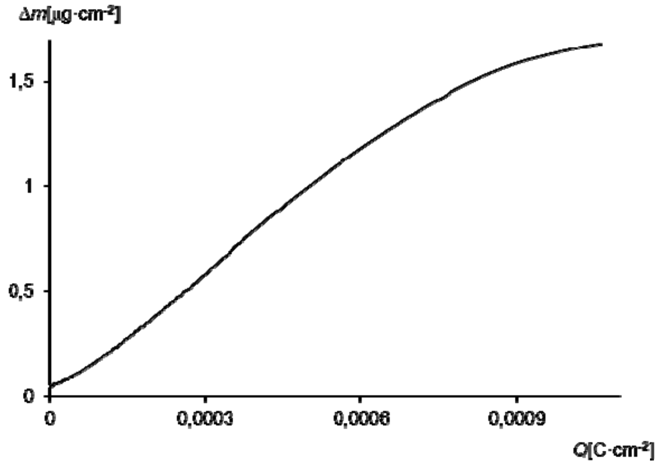

Figure S14. Δm vs Q plot for oxidation process of *poly*[Ni(salcn)] (after electropolymerization: 3 scans, v = 0.5 $V{\cdot}s^{-1}$) in TBAH(0.1 $mol{\cdot}dm^{-3}$)/CH_2Cl_2 at modified Pt/quartz crystal, v = 0.05 $V{\cdot}s^{-1}$, vs. Ag/AgCl.

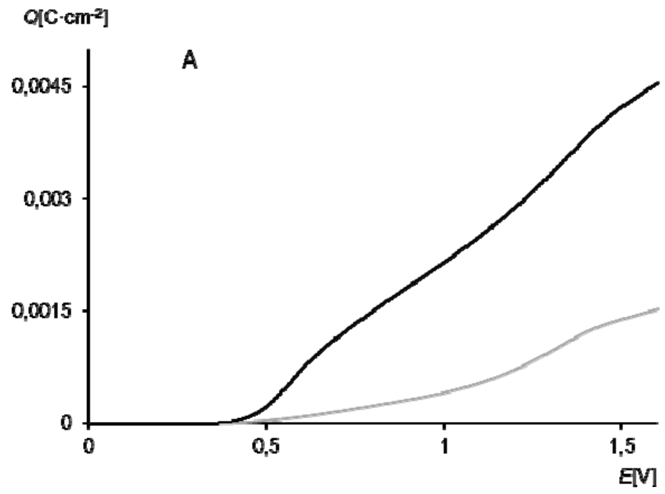

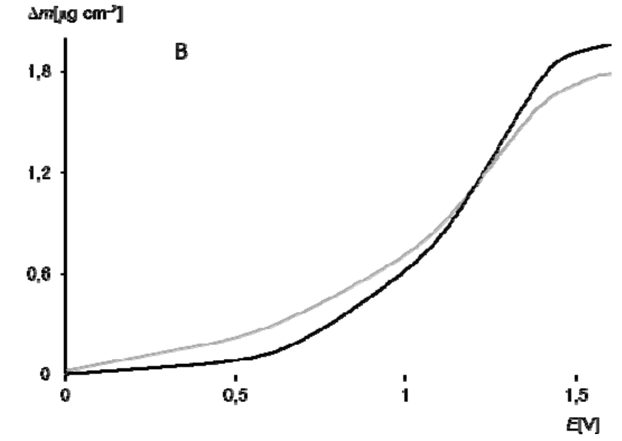

Figure S15. Oxidation process of *poly*[Ni(salcn)] (after electropolymerization: 3 scans, v = 0.4 $V{\cdot}s^{-1}$) at modified Pt/quartz crystal, v = 0.05 $V{\cdot}s^{-1}$, vs. Ag/AgCl; in TBAH(0.1 $mol{\cdot}dm^{-3}$)/CH_2Cl_2—grey lines, in ferrocene(10^{-3} $mol{\cdot}dm^{-3}$)/TBAH/CH_2Cl_2—black lines. (A)—plots Q vs E, (B)—plots Δm vs E.

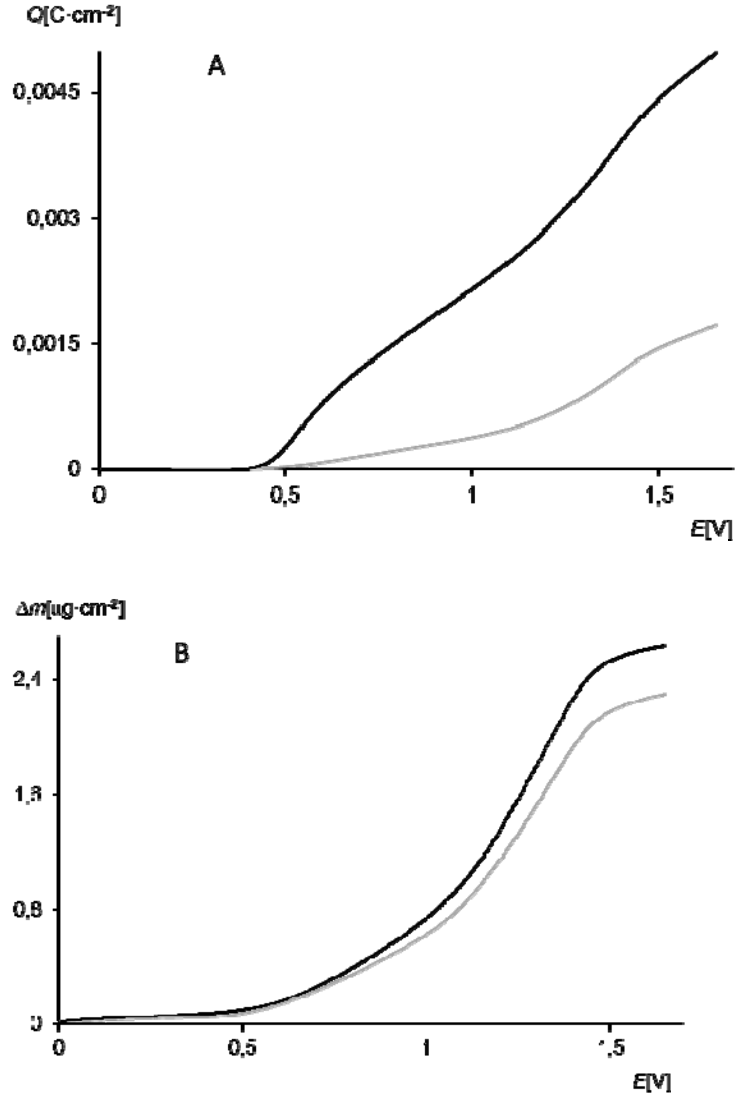

Figure S16. Oxidation process of *poly*[Ni(salcn)] (after electropolymerization: 3 scans, v = 0.3 $V{\cdot}s^{-1}$) at modified Pt/quartz crystal, v = 0.05 $V{\cdot}s^{-1}$, vs. Ag/AgCl; in TBAH(0.1 $mol{\cdot}dm^{-3}$)/CH_2Cl_2—grey lines, in ferrocene(10^{-3} $mol{\cdot}dm^{-3}$)/TBAH/CH_2Cl_2—black lines. (A)—plots Q vs E, (B)—plots Δm vs E.

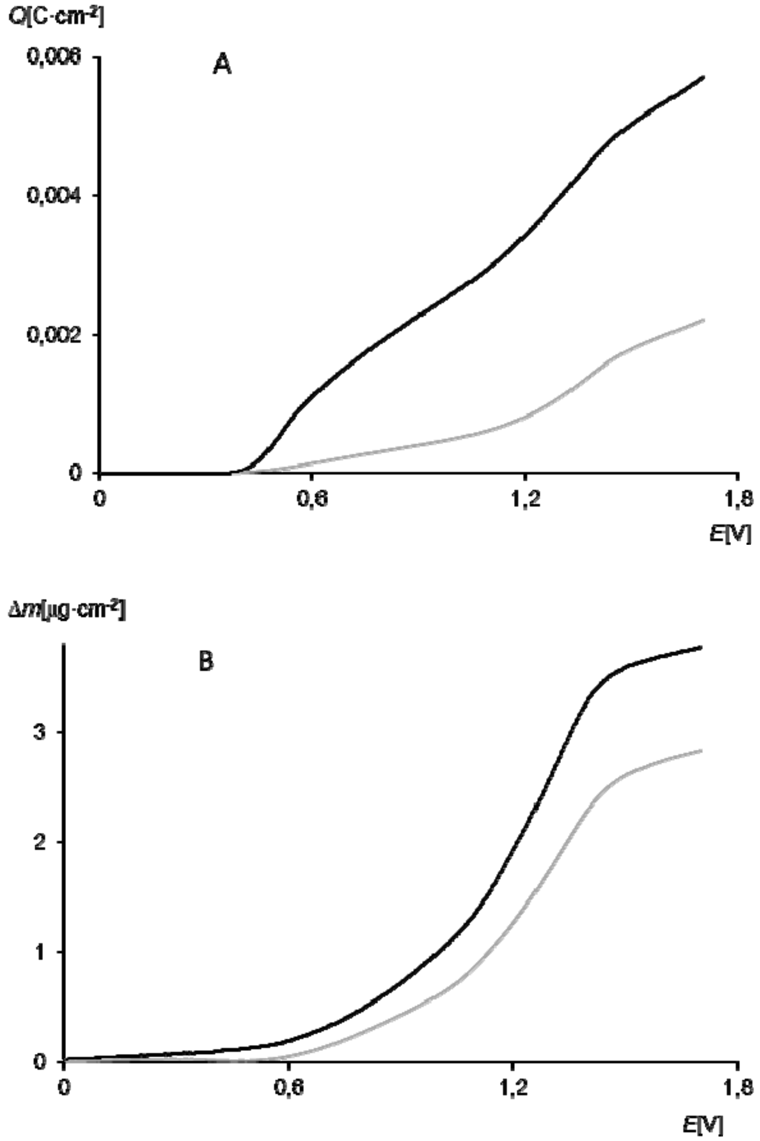

Figure S17. Oxidation process of *poly*[Ni(salcn)] (after electropolymerization: 3 scans, v = 0.2 V·s^{-1}) at modified Pt/quartz crystal, v = 0.05 V·s^{-1}, vs. Ag/AgCl; in TBAH(0.1 mol·dm^{-3})/CH_2Cl_2—grey lines, in ferrocene(10^{-3} mol·dm^{-3})/TBAH/CH_2Cl_2—black lines. (A)—plots Q vs E, (B)—plots Δm vs E.

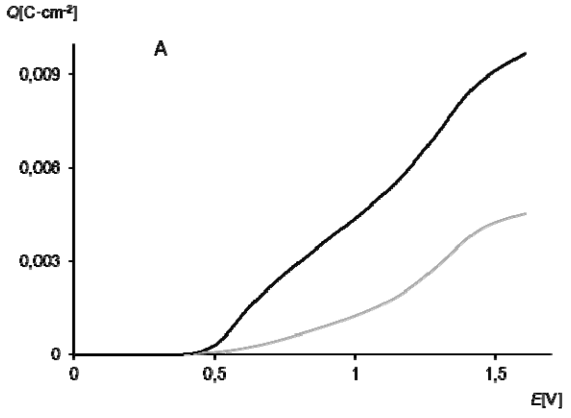

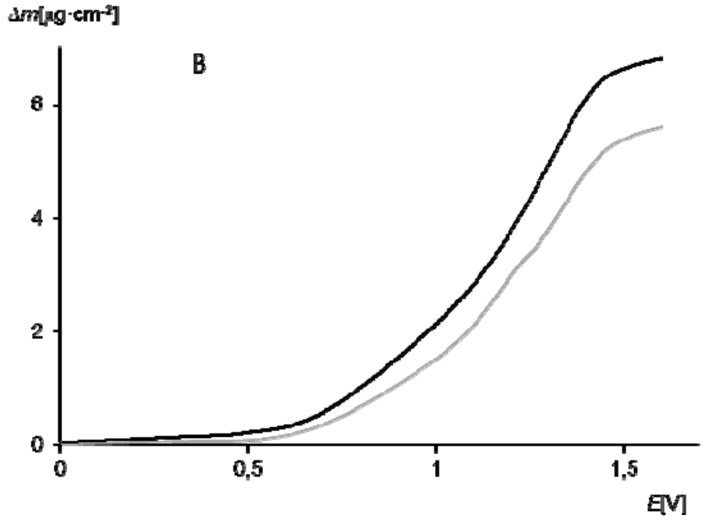

Figure S18. Oxidation process of *poly*[Ni(salcn)] (after electropolymerization: 3 scans, v = 0.05 $V \cdot s^{-1}$) at modified Pt/quartz crystal, v = 0.05 $V \cdot s^{-1}$, vs. Ag/AgCl; in TBAH(0.1 $mol \cdot dm^{-3}$)/CH_2Cl_2—grey lines, in ferrocene(10^{-3} $mol \cdot dm^{-3}$)/TBAH/CH_2Cl_2—black lines. (A)—plots Q vs E, (B)—plots Δm vs E.

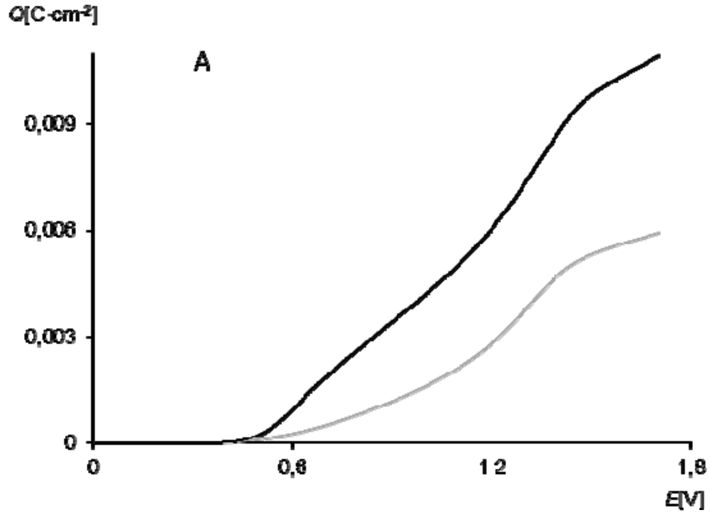

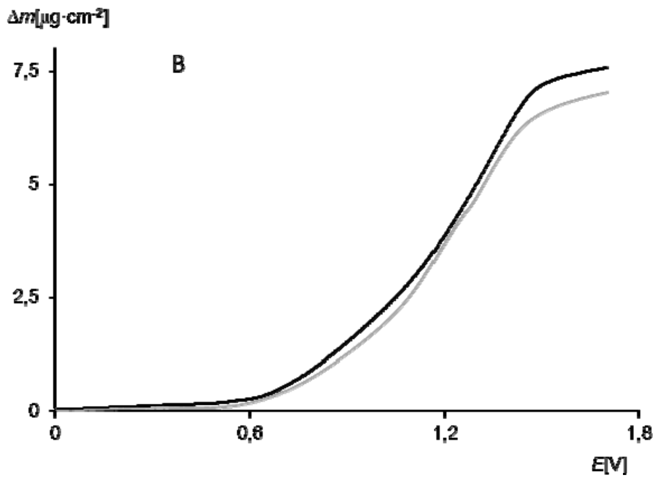

Figure S19. Oxidation process of *poly*[Ni(salcn)] (after electropolymerization: 3 scans, v = 0.04 $V \cdot s^{-1}$) at modified Pt/quartz crystal, v = 0.05 $V \cdot s^{-1}$, vs. Ag/AgCl; in TBAH(0.1 $mol \cdot dm^{-3}$)/CH_2Cl_2—grey lines, in ferrocene(10^{-3} $mol \cdot dm^{-3}$)/TBAH/CH_2Cl_2—black lines. (A)—plots Q vs E, (B)—plots Δm vs E.

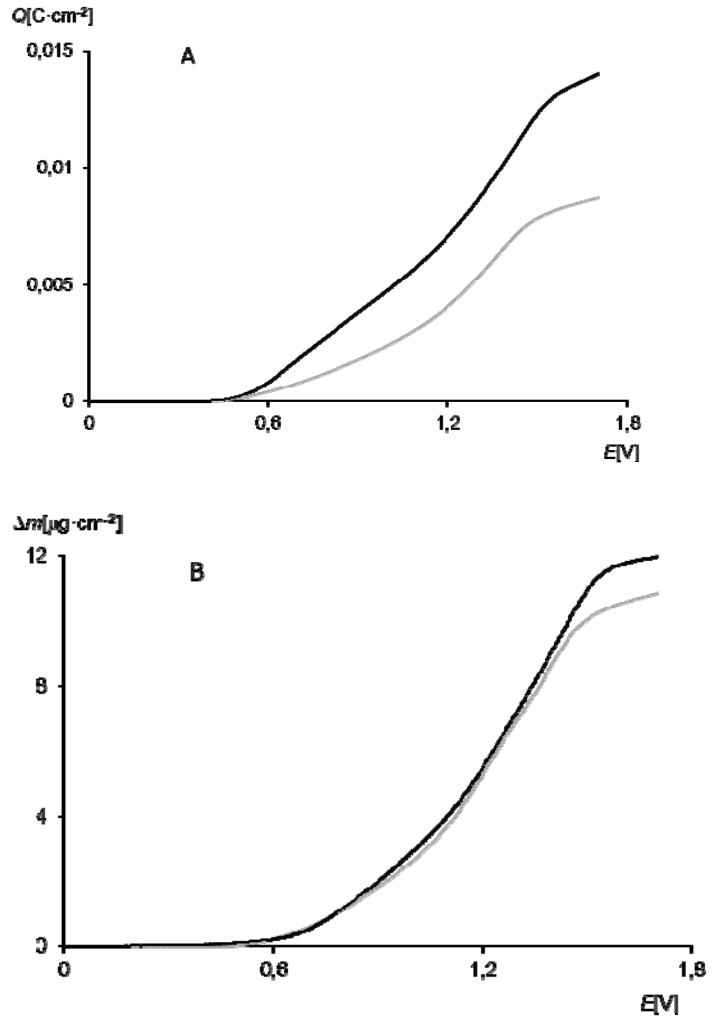

Figure S20. Oxidation process of *poly*[Ni(salcn)] (after electropolymerization: 3 scans, v = 0.02 V·s^{-1}) at modified Pt/quartz crystal, v = 0.05 V·s^{-1}, vs. Ag/AgCl; in TBAH(0.1 mol·dm^{-3})/CH_2Cl_2—grey lines, in ferrocene(10^{-3} mol·dm^{-3})/TBAH/CH_2Cl_2 - black lines. (A)—plots Q vs E, (B)—plots Δm vs E.

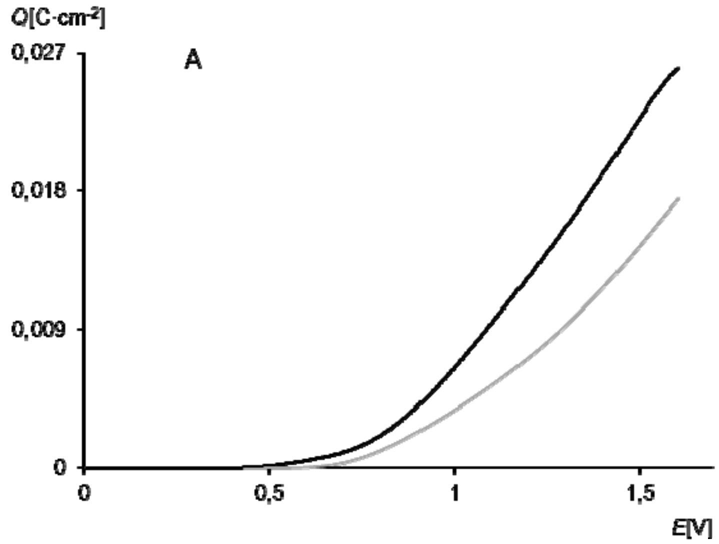

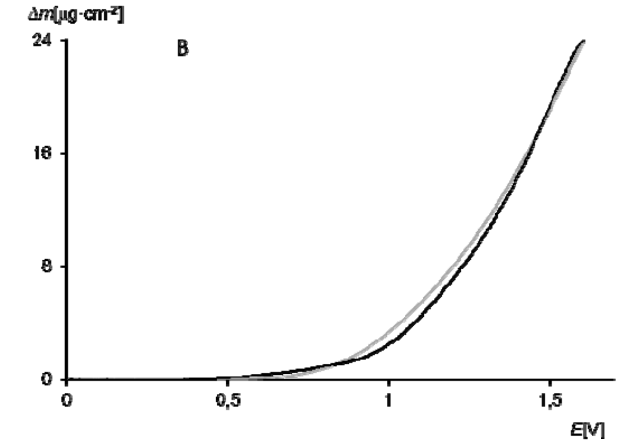

Figure S21. Oxidation process of *poly*[Ni(salcn)] (after electropolymerization: 3 scans, $v = 0.01$ V·s^{-1}) at modified Pt/quartz crystal, $v = 0.05$ V·s^{-1}, vs. Ag/AgCl; in TBAH(0.1 mol·dm^{-3})/CH_2Cl_2—grey lines, in ferrocene(10^{-3} mol·dm^{-3})/TBAH/CH_2Cl_2—black lines. (A)—plots Q vs E, (B)—plots Δm vs E.

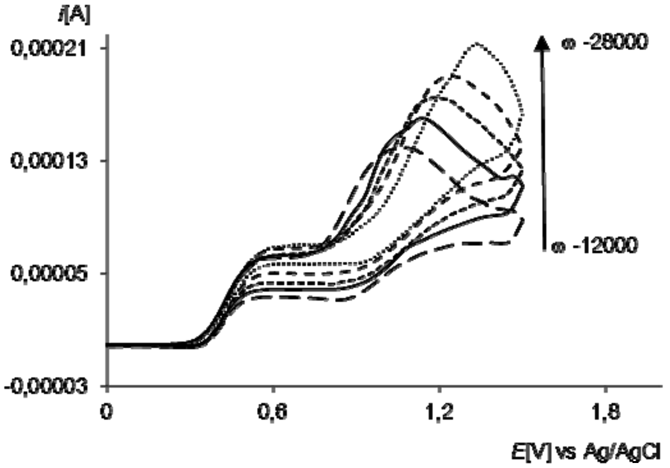

Figure S22. Cyclic voltammograms at rotating Pt*poly*[Ni(salcn)] disc electrode ($\Gamma = 8.51 \cdot 10^{-9}$ mol·cm^{-2}), 2nd scan, $v = 0.05$ V·s-1, *vs.* Ag/AgCl, ω: 1200, 1600, 2000, 2400, 2800 rad$^{1/2}$·s$^{-1/2}$, in ferrocene(10^{-3} mol·dm^{-3})/TBAH(0.1 mol·dm^{-3})/CH_2Cl_2.

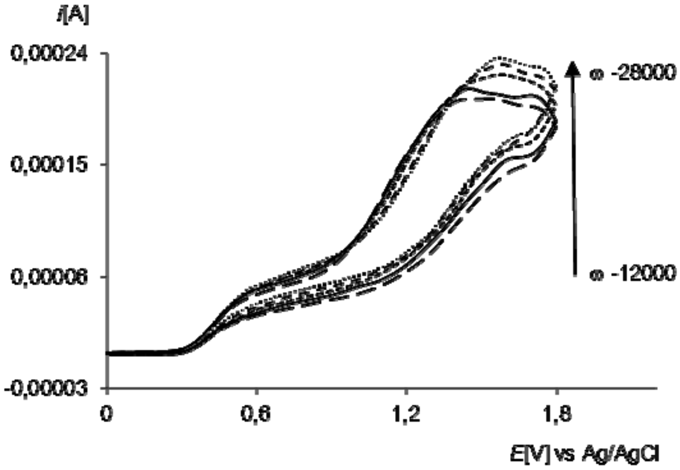

.

Figure S23. Cyclic voltammograms at rotating Pt*poly*[Ni(salcn)] disc electrode (Γ = 12.7·10^{-9} mol·cm^{-2}), 2nd scan, v = 0.05 V·s-1, *vs.* Ag/AgCl, ω: 1200, 1600, 2000, 2400, 2800 rad$^{1/2}$·s$^{-1/2}$, in ferrocene(10^{-3} mol·dm^{-3})/TBAH(0.1 mol·dm^{-3})/CH_2Cl_2.

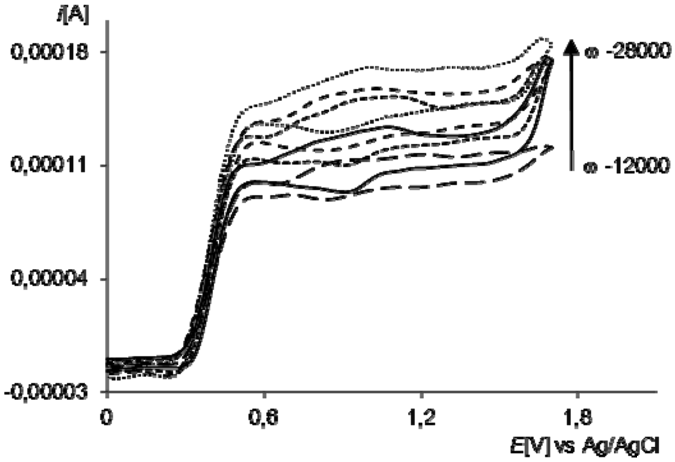

Figure S24. Cyclic voltammograms at rotating Pt*poly*[Ni(salcn)] disc electrode (Γ = 1.22·10^{-9} mol·cm^{-2}), 2nd scan, v = 0.05 V·s-1, *vs.* Ag/AgCl, ω: 1200, 1600, 2000, 2400, 2800 rad$^{1/2}$·s$^{-1/2}$, in ferrocene(10^{-3} mol·dm^{-3})/TBAH(0.1 mol·dm^{-3})/CH_2Cl_2.

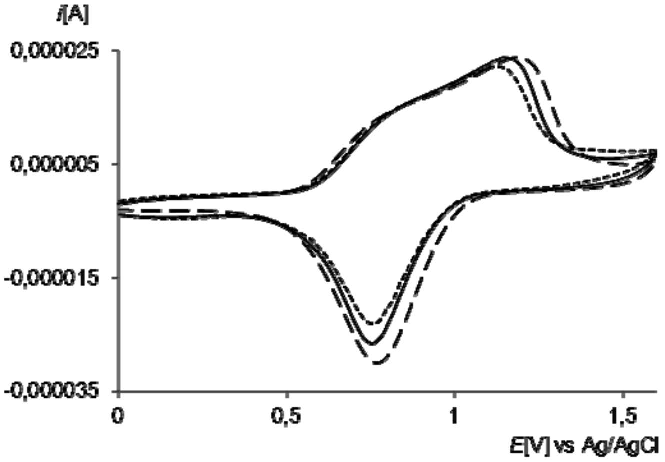

Figure S25. Cyclic voltammograms at rotating Pt*poly*[Ni(salcn)] disc electrode (Γ = 6.27·10^{-9} mol·cm^{-2}), 2nd scan, v = 0.05 V·s^{-1}, *vs.* Ag/AgCl, in TBAH(0.1 mol·dm^{-3})/CH_2Cl_2.; ω = 0—dashed line; ω = 1200—solid line; ω = 2800 rad$^{1/2}$·s$^{-1/2}$—dotted line.

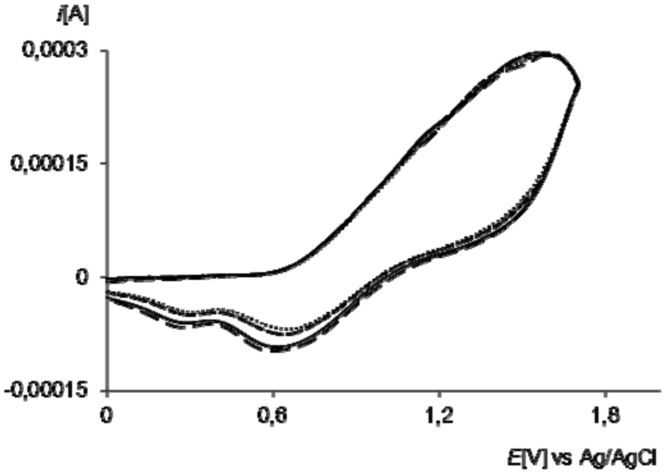

Figure S26. Cyclic voltammograms at rotating Pt*poly*[Ni(salcn)] disc electrode (Γ = 17.1·10^{-9} mol·cm^{-2}), 2nd scan, v = 0.05 V·s^{-1}, *vs.* Ag/AgCl, ω: 1200, 1600, 2000, 2400, 2800 $rad^{1/2}$·$s^{-1/2}$, in ferrocene(10^{-3} mol·dm^{-3})/TBAH(0.1 mol·dm^{-3})/CH_2Cl_2.
